# Supplementary material for: Investigating the role of FOX gene family in development and stress response in Labeo rohita: A multi-faceted analysis of phylogeny and genome characterization
Source: PLoS One. 2025 Aug 21;20(8):e0323740. doi: 10.1371/journal.pone.0323740 (PMC12370200; doi:10.1371/journal.pone.0323740)
Supplement: S1 Table — (DOCX) [file pone.0323740.s001.docx]

**Table S1.** Genomic properties of the FOX gene superfamily in *L. rohita.*

| **Gene Name** | **Gene Symbol** | **Gene Length (nt)** | **Chromosome Length (bp)** | **Gene Location on Chr. (start)** | **Gene Location on Chr. (End)** | **Strand Direction** |
| --- | --- | --- | --- | --- | --- | --- |
| forkhead box A1 | FOXA1 | 2240 | 35622047 | 6450699 | 6452938 | F |
| forkhead box A2 | FOXA2 | 2606 | 35622047 | 32886128 | 32888733 | R |
| forkhead box A3 | FOXA3 | 3819 | 35139235 | 28393641 | 28397459 | F |
| forkhead box C1a | FOXC1 | 6085 | 39672848 | 1862813 | 1868897 | F |
| forkhead box D1 | FOXD1 | 1752 | 46981267 | 24990720 | 24992471 | R |
| forkhead box D3 | FOXD3 | 1781 | 35609041 | 20308840 | 20310620 | R |
| forkhead box F1 | FOXF1 | 5861 | 35139235 | 22958718 | 22964578 | R |
| forkhead box F2a | FOXF2 | 3015 | 39672848 | 1801893 | 1804907 | F |
| forkhead box G1a | FOXG1 | 17550 | 35622047 | 19033219 | 19050768 | R |
| forkhead box H1 | FOXH1 | 4993 | 31394111 | 21633128 | 21638120 | F |
| forkhead box i1 | FOXI1 | 1397 | 31394111 | 3432635 | 3434031 | F |
| forkhead box I2 | FOXI2 | 2927 | 36609346 | 12237663 | 12240589 | R |
| forkhead box L1 | FOXL1 | 2516 | 35139235 | 11077402 | 11079917 | R |
| forkhead box L2a | FOXL2 | 10238 | 38858801 | 10534105 | 10544342 | F |
| forkhead box M1 | FOXM1 | 6522 | 46442965 | 42244898 | 42251419 | F |
| forkhead box O1a | FOXO1 | 41873 | 38858801 | 11331585 | 11373457 | R |
| forkhead box O3b | FOXO3 | 38863 | 33901507 | 20904624 | 20943486 | R |
| forkhead box O4 | FOXO4 | 9247 | 33426740 | 7625375 | 7634621 | F |
| forkhead box P1b | FOXP1 | 207743 | 35609041 | 27032605 | 27240347 | R |
| forkhead box P2 | FOXP2 | 109399 | 46442965 | 41361031 | 41470429 | R |
| forkhead box P3b | FOXP3 | 9893 | 37901974 | 20804872 | 20814764 | R |
