# Supplementary material for: Investigating the role of FOX gene family in development and stress response in Labeo rohita: A multi-faceted analysis of phylogeny and genome characterization
Source: PLoS One. 2025 Aug 21;20(8):e0323740. doi: 10.1371/journal.pone.0323740 (PMC12370200; doi:10.1371/journal.pone.0323740)
Supplement: S1 Fig — (PDF) [file pone.0323740.s002.pdf]

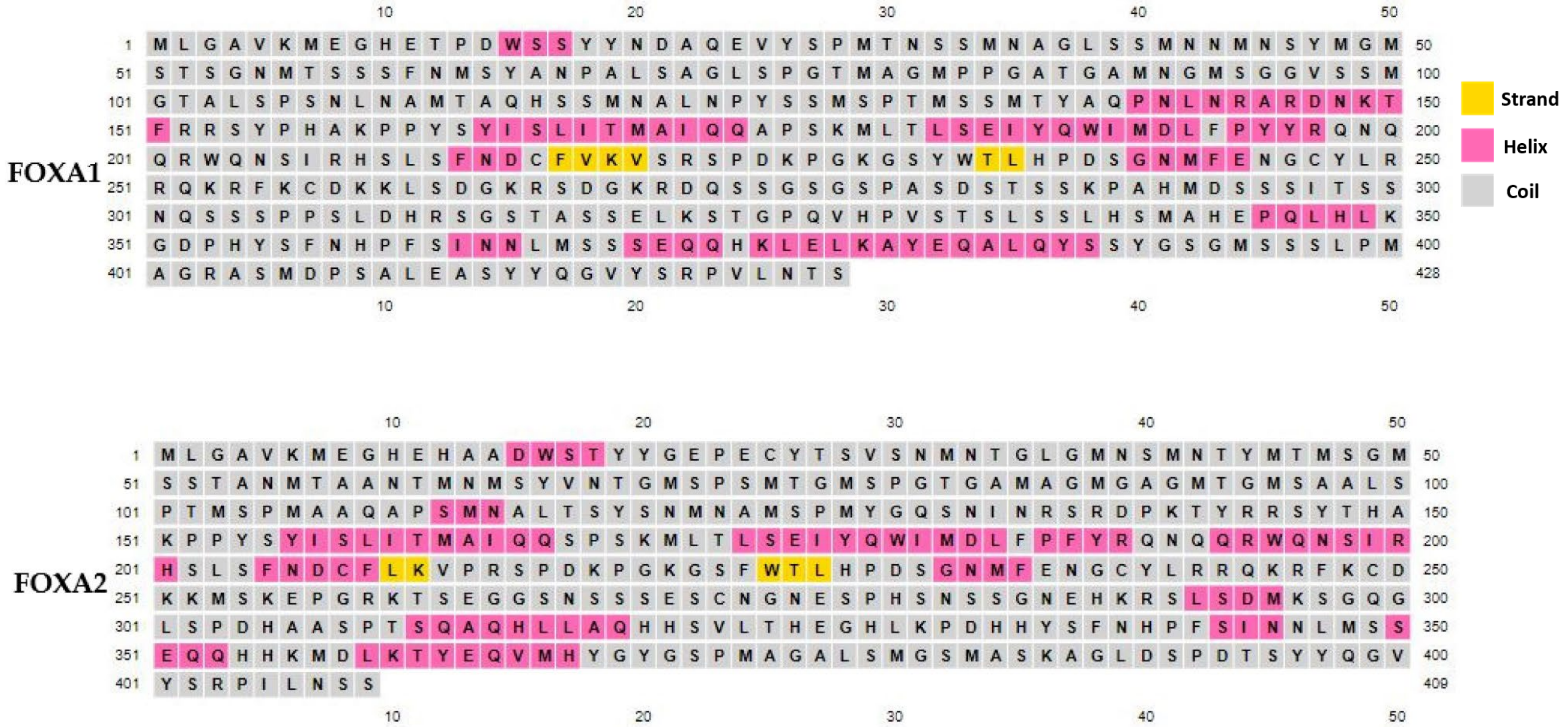
Strand
Helix
Coil
FOXA1
1 M L G A V K M E G H E T P D W S S Y Y N D A Q E V Y S P M T N S S M N A G L S S M N N M N S Y M G M 50
51 S T S G N M T S S S F N M S Y A N P A L S A G L S P G T M A G M P P G A T G A M N G M S G G V S S M 100
101 G T A L S P S N L N A M T A Q H S S M N A L N P Y S S M S P T M S S M T Y A Q P N L N R A R D N K T 150
151 F R R S Y P H A K P P Y S Y I S L I T M A I Q Q A P S K M L T L S E I Y Q W I M D L F P Y Y R Q N Q 200
201 Q R W Q N S I R H S L S F N D C F V K V S R S P D K P G K G S Y W T L H P D S G N M F E N G C Y L R 250
251 R Q K R F K C D K K L S D G K R S D G K R D Q S S G S G S P A S D S T S S K P A H M D S S S I T S S 300
301 N Q S S S P P S L D H R S G S T A S S E L K S T G P Q V H P V S T S L S S L H S M A H E P Q L H L K 350
351 G D P H Y S F N H P F S I N N L M S S S E Q Q H K L E L K A Y E Q A L Q Y S S Y G S G M S S S L P M 400
401 A G R A S M D P S A L E A S Y Y Q G V Y S R P V L N T S 428
FOXA2
1 M L G A V K M E G H E H A A D W S T Y Y G E P E C Y T S V S N M N T G L G M N S M N T Y M T M S G M 50
51 S S T A N M T A A N T M N M S Y V N T G M S P S M T G M S P G T G A M A G M G A G M T G M S A A L S 100
101 P T M S P M A A Q A P S M N A L T S Y S N M N A M S P M Y G Q S N I N R S R D P K T Y R R S Y T H A 150
151 K P P Y S Y I S L I T M A I Q Q S P S K M L T L S E I Y Q W I M D L F P F Y R Q N Q Q R W Q N S I R 200
201 H S L S F N D C F L K V P R S P D K P G K G S F W T L H P D S G N M F E N G C Y L R R Q K R F K C D 250
251 K K M S K E P G R K T S E G G S N S S S E S C N G N E S P H S N S S G N E H K R S L S D M K S G Q G 300
301 L S P D H A A S P T S Q A Q H L L A Q H H S V L T H E G H L K P D H H Y S F N H P F S I N N L M S S 350
351 E Q Q H H K M D L K T Y E Q V M H Y G Y G S P M A G A L S M G S M A S K A G L D S P D T S Y Y Q G V 400
401 Y S R P I L N S S 409

FOXA3

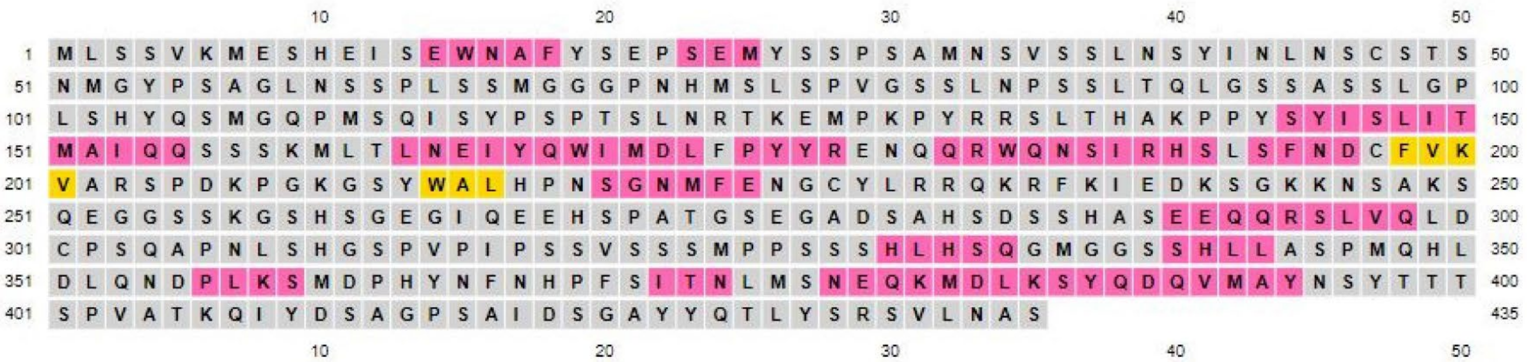
10 20 30 40 50
1 MLSSVKMESHEISEWNAFYSEPSEMYSSPSAMNSVSSLNSYINLNSCSTS 50
51 NMGYPSAGLNSSPLSSMGGGPNHMSLSPVGSSLNPSSLTQLGSSASSLGP 100
101 LSHYQSMGQPMSQISYPSPTSLNRTKEMPKPYRRSLTHAKPPYSYISLIT 150
151 MAIQQSSSKMLTLNEIYQWIMDLFPYYRENQQRWQNSIRHSLSFNDCFVK 200
201 VARSPDKPGKGSYWALHPNSGNMFENGCYLRRQKRFKIEDKSGKKNSAKS 250
251 QEGGSSKGSHSGEGIQEEHSPATGSEGADSAHSDSSHASEEQQRSLVQLD 300
301 CPSQAPNLSHGSPVPIPSSVSSSMPPSSSHLHSQGMGGSSHLLASPMQHL 350
351 DLQNDPLKSMDPHYNFNHPFSITNLMSNEQKMDLKSYQDQVMAYNSYTTT 400
401 SPVATKQIYDSAGPSAIDSGAYYQTLYSRSVLNAS 435
10 20 30 40 50

FOXC1

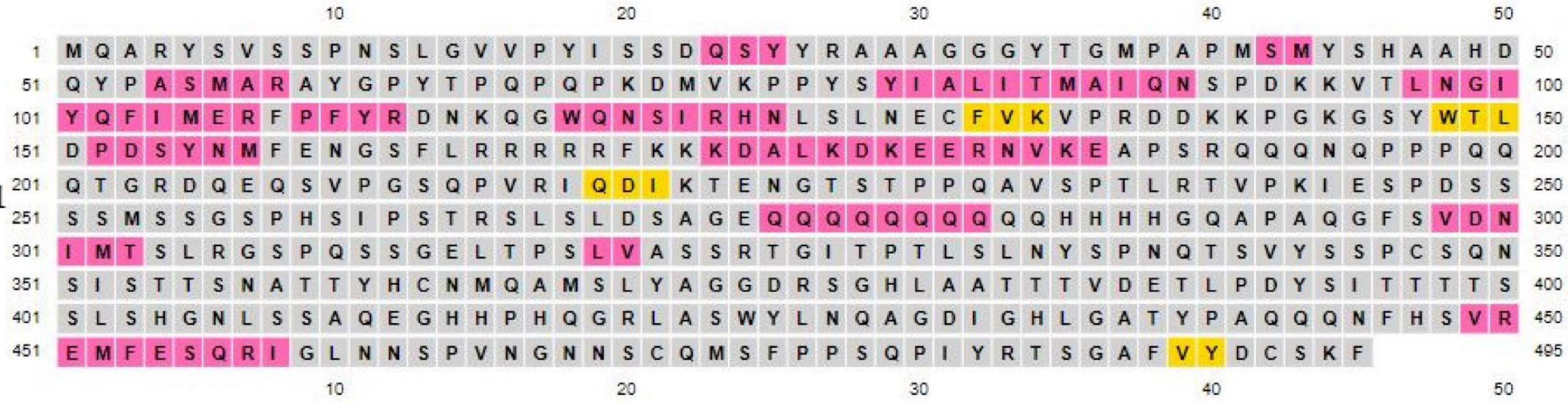
10 20 30 40 50
1 MQARYSVSSPNSLGVVPYISSDQSYYRAAAGGGYTGMPAPMSMYSHAAHD 50
51 QYPASMARAYGPYTPQPQPKDMVKPPYSYIALITMAIQNSPDKKVTLNGI 100
101 YQFIMERFPFYRDNKQGWQNSIRHNLSLNECFVKVPRDDKKPGKGSYWTL 150
151 DPDSYNMFENGSFLRRRRRFKKKDALKDKEERNVKEAPSRQQQNQPPPQQ 200
201 QTGRDQEQSVPGSQPVRIQDIKTENGTSTPPQAVSPTLRTVPKIESPDSS 250
251 SSMSSGSPHSIPSTRSLSLDSAGEQQQQQQQQQQHHHHGQAPAQGFSVDN 300
301 IMTSLRGSPQSSGELTPSLVASSRTGITPTLSLNYSPNQTSVYSSPCSQN 350
351 SISTTSNATTYHCNMQAMSLYAGGDRSGHLAATTTVDETLPDYSITTTTS 400
401 SLSHGNLSSAQEGHHPHQGRLASWYLNQAGDIGHLGATYPAQQQNFHSVR 450
451 EMFESQRIGLNNSPVNGNNSCQMSFPPSQPIYRTSGAFVYDCSKF 495
10 20 30 40 50

FOXD1

1 MTLSSEMSDASVLSEETDIDVVGEGDDGDGHTRSYVDEVAQMHDEILLSG 50
51 SPSCVDASAARDPYKPASKNTLVKPPYSYIALITMAILQSPKKRLTLSEI 100
101 CDFISNRFPYYREKFPAWQNSIRHNLSLNDCFVKIPREPGNPGKGNYWTL 150
151 DPESADMFDNGSFLRRRKRFKRQQAPELLREHGGFLPAAAAYGYGPYGCG 200
201 YGLQLQSYHAHSALLAFQQQQQQQPPPPSSRHPHTGTLIPAPSLMPTTTE 250
251 LARSRFYPPLSPGLSSSIQTAAKSPVHRSPFSIDSIIGSSLSPTHSHCAS 300
301 RTSPVVPVLPPTLASQHSPTPLPGVLHGPTSLHETYANRIVSGTSGC 347

FOXD3

1 MTLSGGTSASNMSGQTVLTADDVDIDVVGEGDEGMERDSDCESQCMQDRG 50
51 DEVEEIEVKERSDSPCESAGEGESKGDAQESSSGPMQSKPKSSLVKPPYS 100
101 YIALITMAILQSPQKKLTLSGICEFISNRFPYYREKFPAWQNSIRHNLSL 150
151 NDCFVKIPREPGNPGKGNYWTLDPQSEDMFDNGSFLRRRKRFKRHQPDIL 200
201 RDQTALMMQSFGAYSIGNPYGRHYGIHPAAYSHPAAALQYPYIPPVGPML 250
251 PPAVPLLPSAELNRKAFNSQLSPSLQLQLNSLSTASIIKSEPSSRPSFSI 300
301 ENIIGVSSSSSSAQTFLRPPVTVQSALLSAQSLSLTRTSAAIAPILSVPS 350
351 NIISGQFLPTASTAAVSKWPSQ 372

FOXF1

1 MTAEVQQPSVQTPAHSSPMSEKPHGQTPVMETSSSSSSTKTKKTNAGIRR 50
51 PEKPPYSYIALIVMAIQSSPTKRLTLSEIYQFLQSRFPFFRGSYQGWKNS 100
101 VRHNLSLNECFIKLPKGLGRPGKGHYWTIDPASEFMFEEGSFRRRPRGFR 150
151 RKCQALKPSMYSMMNGLGFNHIPESYNFQGGGGGLSCPPNSLPLESGIGM 200
201 MNGHLASNMEGMGLAGHSMSHLSTNSGHSYMGSCTGSSGSEYPHHDNSAS 250
251 PLLASGGVMEPHAVYSSTASAWPPAPPASLNNGASYIKQQPLSPCNPGAN 300
301 PLQPSLPTHSLEQSYLHQNGHGTTDLQGIPRYHSQSPSMCDRKEFVFSFN 350
351 AMTSSSMHSPGSSSYYHHQQVSYQDIKPCVM 381

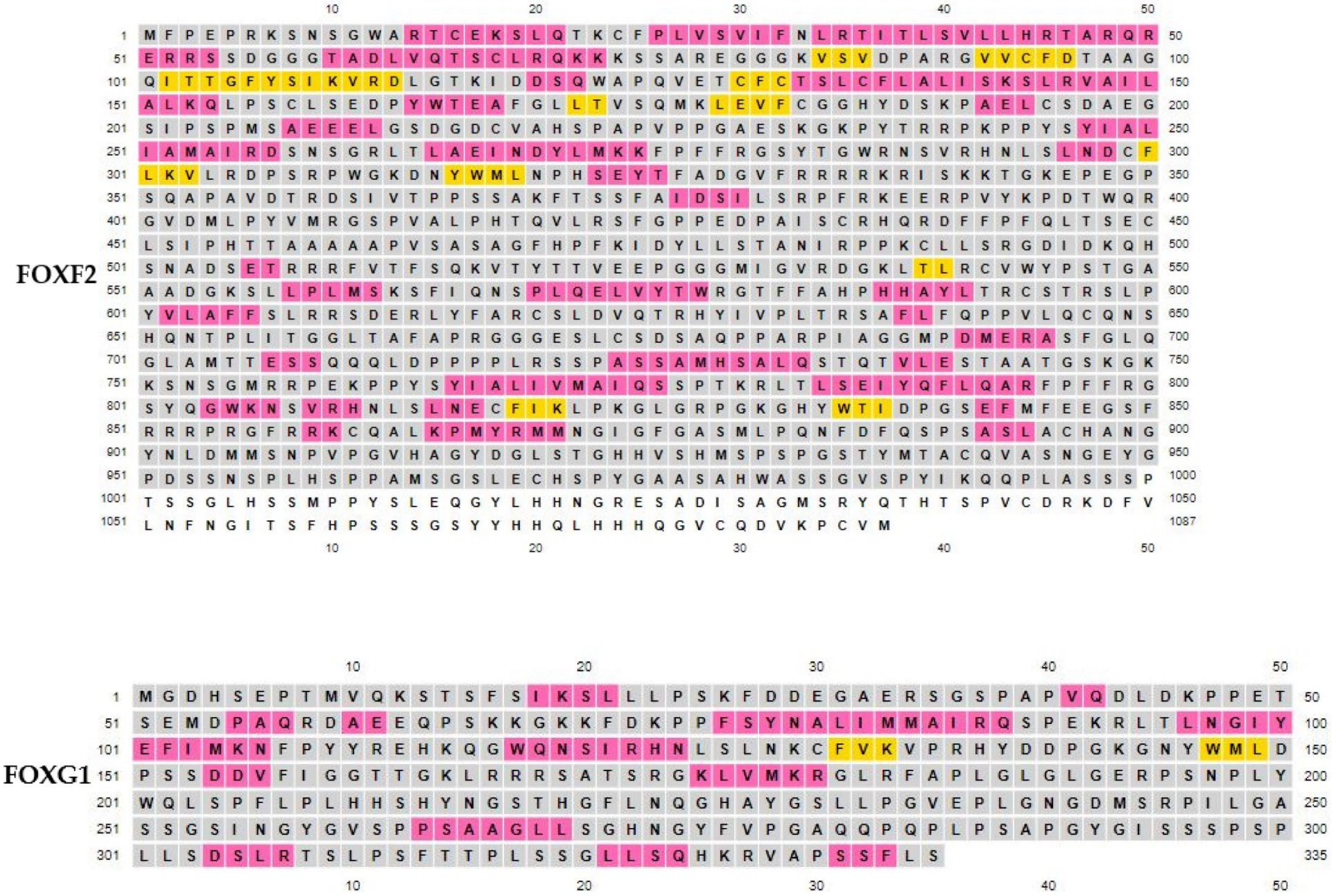
FOXF2
FOXG1

FOXH1

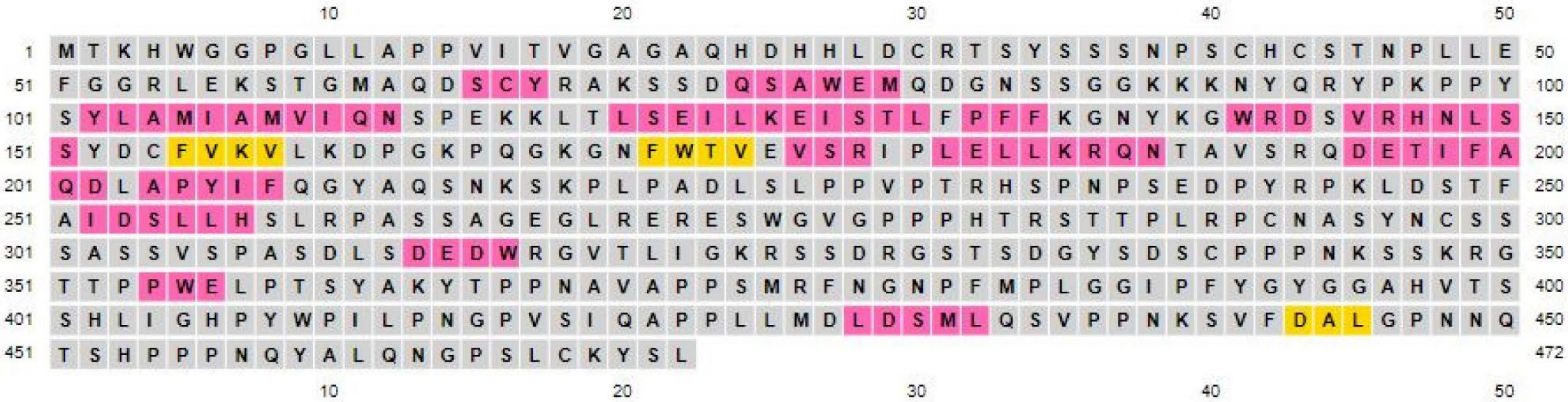
10 20 30 40 50
1 MTKHWGGPGLLAPPVITVGAGAQHDHHLDCRTSYSSSNPSCHCSTNPLLE 50
51 FGGRLEKSTGMAQDSCYRAKSSDQSAWEMQDGNSSGGKKKNYQRYPKPPY 100
101 SYLAMIAMVIQNSPEKKLTLSEILKEISTLFPFFKGNYKGWRDSVRHNLS 150
151 SYDCFVKVLKDPGKPQGKGNFWTVEVSRIPLELLKRQNTAVSRQDETIFA 200
201 QDLAPYIFQGYAQSNKSKPLPADLSLPPVPTRHSPNPSEDPYRPKLDSTF 250
251 AIDSLLHSLRPASSAGEGLRERESWGVGPPPHTRSTTPLRPCNASYNCSS 300
301 SASSVSPASDLSDEDWRGVTLIGKRSSDRGSTSDGYSDSCPPPNKSSKRG 350
351 TTPPWELPTSYAKYTPPNAVAPPSMRFNGNPFMPLGGIPFYGYGGAHVTS 400
401 SHLIGHPYWPILPNGPVSIQAPPLLMDLDSMLQSVPPNKSVFDALGPNNQ 450
451 TSHPPPNQYALQNGPSLCKYSL 472
10 20 30 40 50

FOXI1

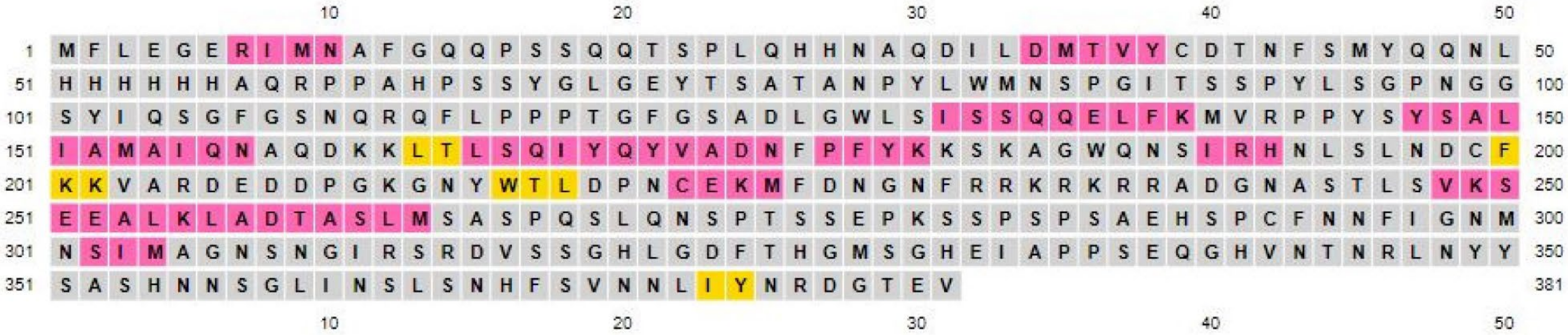
10 20 30 40 50
1 MFLEGERIMNAFGQQPSSQQTSPLQHHNAQDILDMTVYCDTNFSMYQQNL 50
51 HHHHHHAQRPPAHPSSYGLGEYTSATANPYLWMNSPGITSSPYLSGPNGG 100
101 SYIQSGFGSNQRQFLPPPTGFGSADLGWLSISSQQELFKMVRPPYSYSAL 150
151 IAMAIQNAQDKKLTLSQIYQYVADNFPFYKKSKAGWQNSIRHNLSLNDCF 200
201 KKVARDEDDPGKGNYWTLDPNCEKMFDNGNFRRKRKRRADGNASTLSVKS 250
251 EEALKLADTASLMSASPQSLQNSPTSSEPKSSPSPSAEHSPCFNNFIGNM 300
301 NSIMAGNSNGIRSRDVSSGHLGDFTHGMSGHEIAPPSEQGHVNTNRLNYY 350
351 SASHNNSGLINSLSNHFSVNNLIYNRDGTEV 381
10 20 30 40 50

FOXI2

```
1    M N T I D A Q I H N N S N A A V N P L Q Q L P K S A H E T S D M A V Y C D N F S V Y H Q Q S L P A A   50
51   Q R P A G Y G L G D Y A T P N P Y L W L N G P S V N S S S S Y I H G N N S A S F I P P A Y G S Q R Q   100
101  Y L T N S S G F A G P D L G W L S I A S Q E E L L K L V R P P Y S Y S A L I A M A I Q N A H E K K L   150
151  T L S Q I Y Q Y V A D N F P F Y K K S K A G W Q N S I R H N L S L N D C F K K V P R D E D D P G K G   200
201  N Y W T L D P N C E K M F D N G N F R R K R K R R S D S S T G V A S N T K P E D D R Q L A G I K P T   250
251  D S P H L T G P A S P D T D A A N D S H K G A S P A G L S S A P C F N N F F N S M S A L S S S S A P   300
301  T S R Q G S L G L V N E L S S R N I S A L S P Y H A S S A P D A G G A A E L Q D S V H V N R G M Y Y   350
351  N S F T G G Q S A Q F N G H F Y N S F S V N S L I Y P R D G T E L   383
```

FOXL1

```
1    M S L Y H S S V P Q A A S A L T L S N S S L I Y V Y G G E V G G I I P A L G F A S S R Q E P P Q K P   50
51   P Y S Y I A L I A M A I K N S P D K R A T L S G I Y Q F I M D R F P Y Y H D N K Q G W Q N S I R H N   100
101  L S L N D C F I K V P R E K G R P G K G S Y W T L D T K C L D M F E N G N Y R R R K R K C R T Q E T   150
151  G E T K M G H K R T R A L N S K L H Q S A H L E K E S P L K D T D K R E K H P E R D V N I Q P Q N N   200
201  N A S D S T K D W C S A P S T T A G T N P R C P T P E Q T S A V S L N A S L N A R E T P V P A M H D   250
251  T G N A Q T G D A K S K E T N A Q T H P R K T T D K S K E F S I D S I L S K K E N Q F Q R R C G V A   300
301  G G A P V T C L E S R G Y A L S S S L I A H A H P Q L Y P R G F P L C S Y L S L T C P D K I L N F R   350
351  E K T D D K L F F   359
```

FOXL2

```
1    M A S Y H S L D D D A M A L M V H D T N A V K K E E F A K D E P V Q E P S S E K V D P S Q K P P Y S   50
51   Y V A L I A M A I R E S S E K R L T L S G I Y Q Y I I T K F P F Y E K N K K G W Q N S I R H N L S L   100
101  N E C F I K V P R E G G G E R K G N Y W T L D P A C E D M F E K G N Y R R R R R M K R P F R P P A A   150
151  H F Q P G K S L F G G D G Y G S Y L P A P K Y L Q S G F M N N S W P L A Q P P P P M S Y A S C Q M A   200
201  N G N M G P M K G L S A P S Y N P Y S R M Q A M G L P N M M N S Y S G M G H H Q H Q A Q Q Q Q P S P   250
251  V P S N S A A A L Q F T C S R Q P A E L Y S Y W D H E A K N S S L H S R I D I   289
```

FOXM1

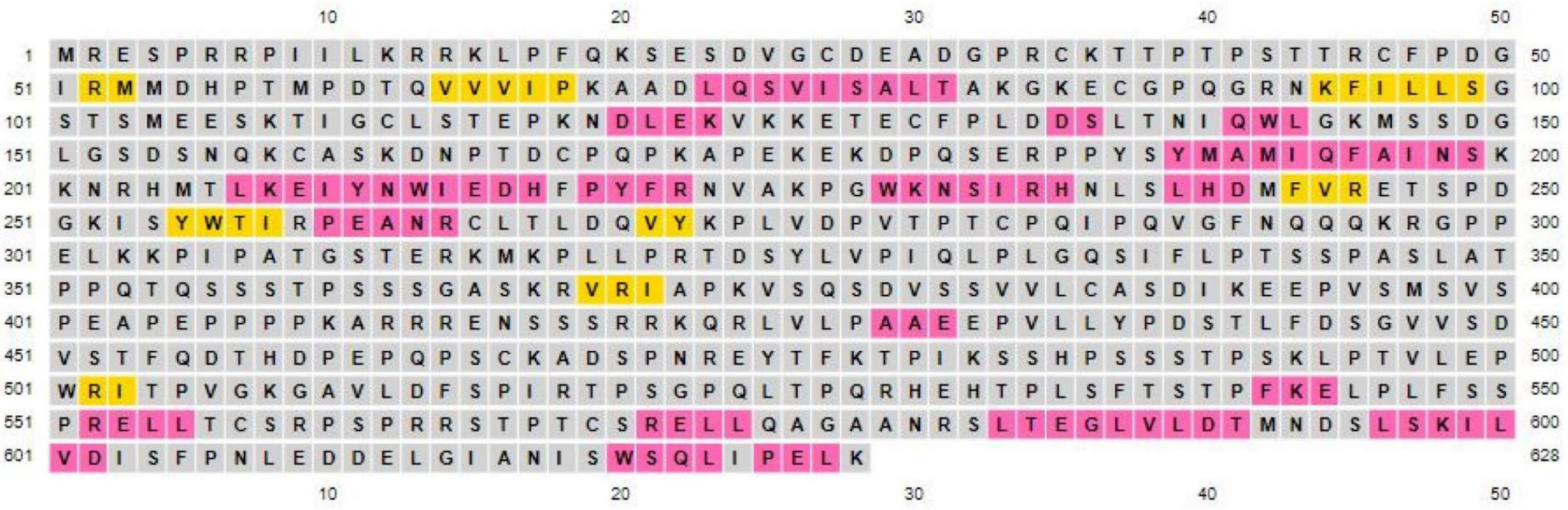
10 20 30 40 50
1 M R E S P R R P I I L K R R K L P F Q K S E S D V G C D E A D G P R C K T T P T P S T T R C F P D G 50
51 I R M M D H P T M P D T Q V V V I P K A A D L Q S V I S A L T A K G K E C G P Q G R N K F I L L S G 100
101 S T S M E E S K T I G C L S T E P K N D L E K V K K E T E C F P L D D S L T N I Q W L G K M S S D G 150
151 L G S D S N Q K C A S K D N P T D C P Q P K A P E K E K D P Q S E R P P Y S Y M A M I Q F A I N S K 200
201 K N R H M T L K E I Y N W I E D H F P Y F R N V A K P G W K N S I R H N L S L H D M F V R E T S P D 250
251 G K I S Y W T I R P E A N R C L T L D Q V Y K P L V D P V T P T C P Q I P Q V G F N Q Q Q K R G P P 300
301 E L K K P I P A T G S T E R K M K P L L P R T D S Y L V P I Q L P L G Q S I F L P T S S P A S L A T 350
351 P P Q T Q S S S T P S S S G A S K R V R I A P K V S Q S D V S S V V L C A S D I K E E P V S M S V S 400
401 P E A P E P P P P K A R R R E N S S S R R K Q R L V L P A A E E P V L L Y P D S T L F D S G V V S D 450
451 V S T F Q D T H D P E P Q P S C K A D S P N R E Y T F K T P I K S S H P S S S T P S K L P T V L E P 500
501 W R I T P V G K G A V L D F S P I R T P S G P Q L T P Q R H E H T P L S F T S T P F K E L P L F S S 550
551 P R E L L T C S R P S P R R S T P T C S R E L L Q A G A A N R S L T E G L V L D T M N D S L S K I L 600
601 V D I S F P N L E D D E L G I A N I S W S Q L I P E L K 628
10 20 30 40 50

FOXO1

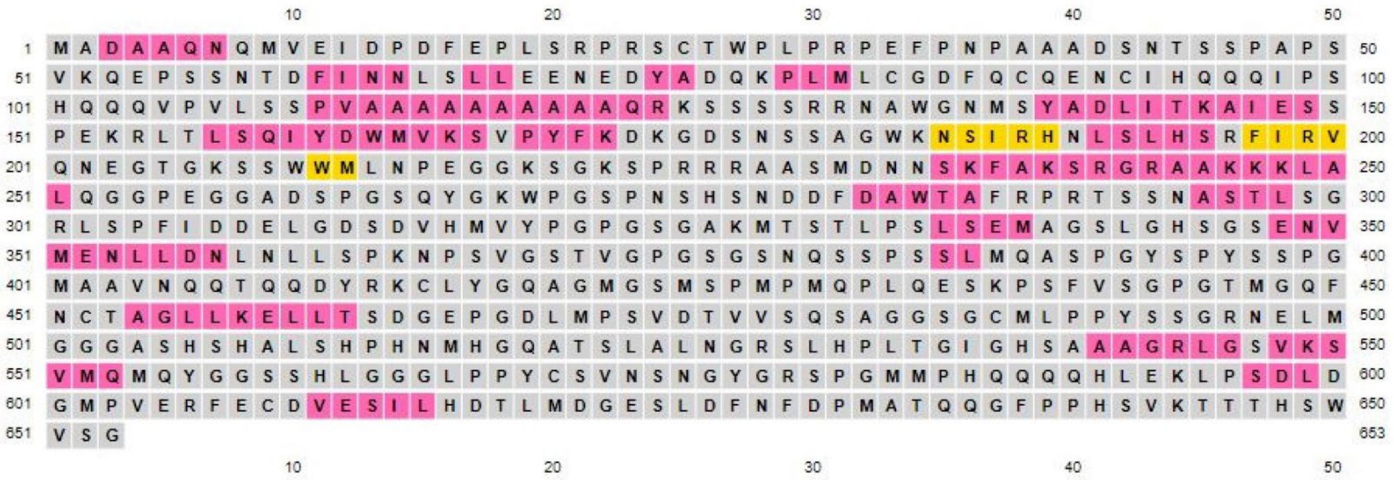
10 20 30 40 50
1 M A D A A Q N Q M V E I D P D F E P L S R P R S C T W P L P R P E F P N P A A A D S N T S S P A P S 50
51 V K Q E P S S N T D F I N N L S L L E E N E D Y A D Q K P L M L C G D F Q C Q E N C I H Q Q Q I P S 100
101 H Q Q Q V P V L S S P V A A A A A A A A A A Q R K S S S S R R N A W G N M S Y A D L I T K A I E S S 150
151 P E K R L T L S Q I Y D W M V K S V P Y F K D K G D S N S S A G W K N S I R H N L S L H S R F I R V 200
201 Q N E G T G K S S W W M L N P E G G K S G K S P R R R A A S M D N N S K F A K S R G R A A K K K L A 250
251 L Q G G P E G G A D S P G S Q Y G K W P G S P N S H S N D D F D A W T A F R P R T S S N A S T L S G 300
301 R L S P F I D D E L G D S D V H M V Y P G P G S G A K M T S T L P S L S E M A G S L G H S G S E N V 350
351 M E N L L D N L N L L S P K N P S V G S T V G P G S G S N Q S S P S S L M Q A S P G Y S P Y S S P G 400
401 M A A V N Q Q T Q Q D Y R K C L Y G Q A G M G S M S P M P M Q P L Q E S K P S F V S G P G T M G Q F 450
451 N C T A G L L K E L L T S D G E P G D L M P S V D T V V S Q S A G G S G C M L P P Y S S G R N E L M 500
501 G G G A S H S H A L S H P H N M H G Q A T S L A L N G R S L H P L T G I G H S A A A G R L G S V K S 550
551 V M Q M Q Y G G S S H L G G G L P P Y C S V N S N G Y G R S P G M M P H Q Q Q Q H L E K L P S D L D 600
601 G M P V E R F E C D V E S I L H D T L M D G E S L D F N F D P M A T Q Q G F P P H S V K T T T H S W 650
651 V S G 653
10 20 30 40 50

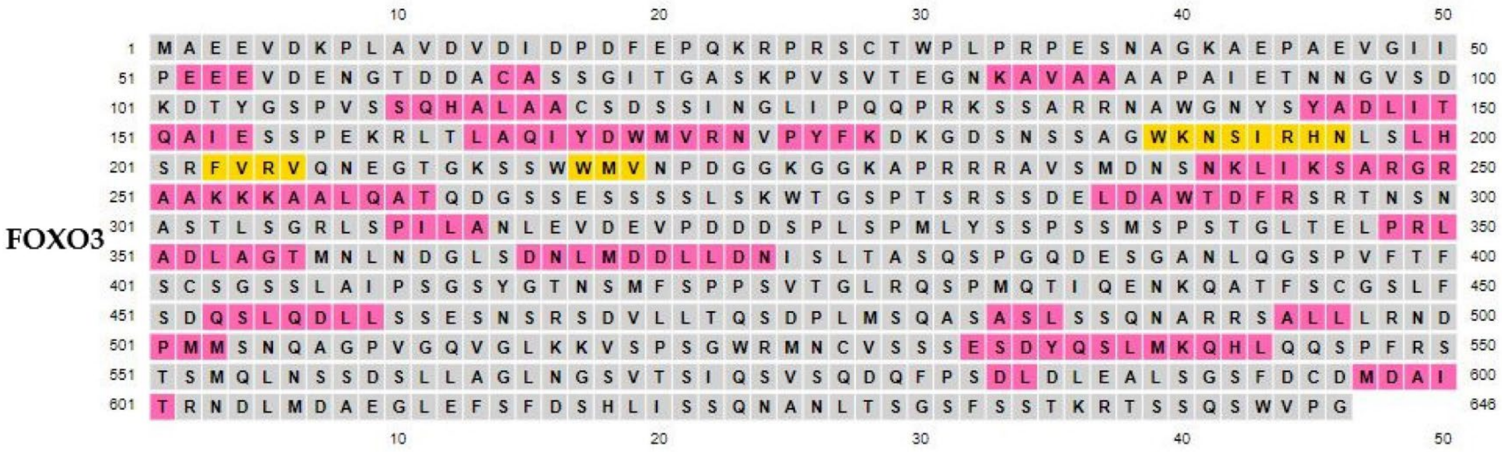
FOXO3
10 20 30 40 50
1 MAEEVDKPLAVDVDIDPDFEPQKRPRSCTWPLPRPESNAGKAEPAEVGII 50
51 PEEEVDENGTDDACASSGITGASKPVSVTEGNKAVAAAAPAIETNNGVSD 100
101 KDTYGSPVSSQHALAACSDSSINGLIPQQPRKSSARRNAWGNYSYADLIT 150
151 QAIESSPEKRLTLAQIYDWMVRNVPYFKDKGDSNSSAGWKNSIRHNLSLH 200
201 SRFVRVQNEGTGKSSWWMVNPDGGKGGKAPRRRAVSMDNSNKLIKSARGR 250
251 AAKKKAALQATQDGSSESSSSLSKWTGSPTSRSSDELDAWTDFRSRTNSN 300
301 ASTLSGRLSPILANLEVDEVPDDDSPLSPMLYSSPSSMSPSTGLTELPRL 350
351 ADLAGTMNLNDGLSDNLMDDLLDNISLTASQSPGQDESGANLQGSPVFTF 400
401 SCSGSSLAIPSGSYGTNSMFSPPSVTGLRQSPMQTIQENKQATFSCGSLF 450
451 SDQSLQDLLSSESNSRSDVLLTQSDPLMSQASASLSSQNARRSALLLRND 500
501 PMMSNQAGPVGQVGLKKVSPSGWRMNCVSSSESDYQSLMKQHLQQSPFRS 550
551 TSMQLNSSDSLLAGLNGSVTSIQSVSQDQFPSDLDLEALSGSFDCDMDAI 600
601 TRNDLMDAEGLEFSFDSHLISSQNANLTSGSFSSTKRTSSQSWVPG 646
10 20 30 40 50

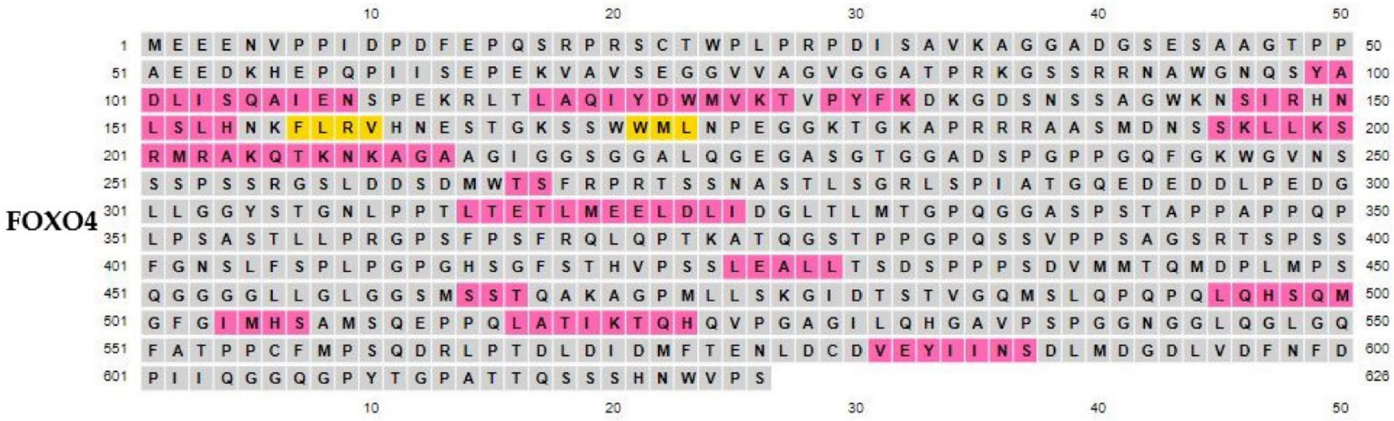
FOXO4
10 20 30 40 50
1 MEEENVPPIDPDFEPQSRPRSCTWPLPRPDISAVKAGGADGSESAAGTPP 50
51 AEEDKHEPQPIISEPEKVAVSEGGVVAGVGGATPRKGSSRRNAWGNQSYA 100
101 DLISQAIENSPEKRLTLAQIYDWMVKTVPYFKDKGDSNSSAGWKNSIRHN 150
151 LSLHNKFLRVHNESTGKSSWWMLNPEGGKTGKAPRRRAASMDNSSKLLKS 200
201 RMRAKQTKNKAGAAGIGGSGGALQGEGASGTGGADSPGPPGQFGKWGVNS 250
251 SSPSSRGSLDDSDMWTSFRPRTSSNASTLSGRLSPIATGQEDEDDLPEDG 300
301 LLGGYSTGNLPPTLTETLMEELDLIDGLTLMTGPQGGASPSTAPPAPPQP 350
351 LPSASTLLPRGPSFPSFRQLQPTKATQGSTPPGPQSSVPPSAGSRTSPSS 400
401 FGNSLFSPLPGPGHSGFSTHVPSSLEALLTSDSPPPSDVMMTQMDPLMPS 450
451 QGGGGLLGLGGSMSSTQAKAGPMLLSKGIDTSTVGQMSLQPQPQLQHSQM 500
501 GFGIMHSAMSQEPPQLATIKTQHQVPGAGILQHGAVPSPGGNGGLQGLGQ 550
551 FATPPCFMPSQDRLPTDLDIDMFTENLDCDVEYIINSDLMDGDLVDFNFD 600
601 PIIQGGQGPYTGPATTQSSSHNWVPS 626
10 20 30 40 50

FOXP1

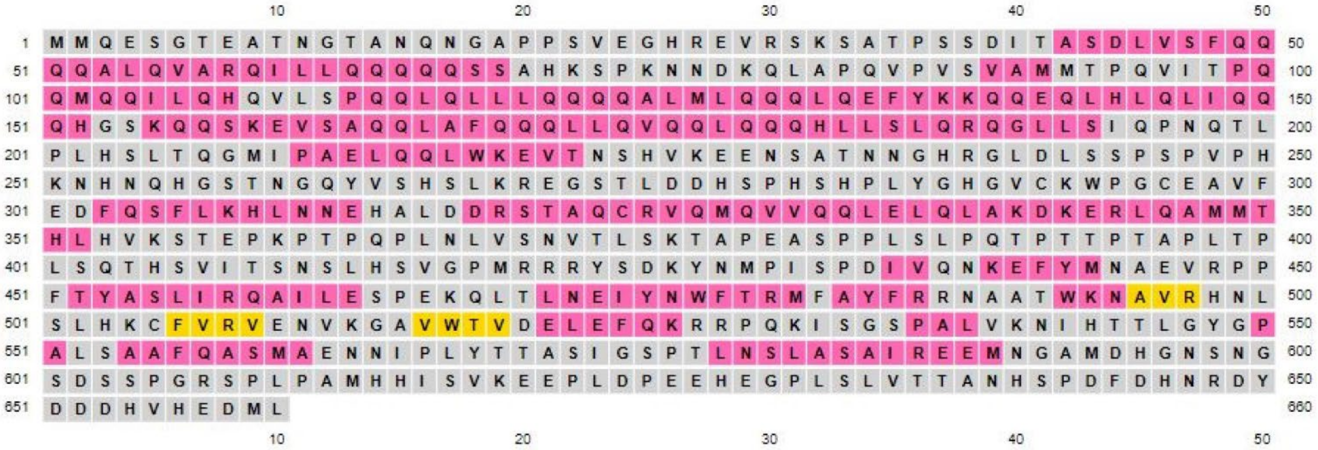

FOXP2

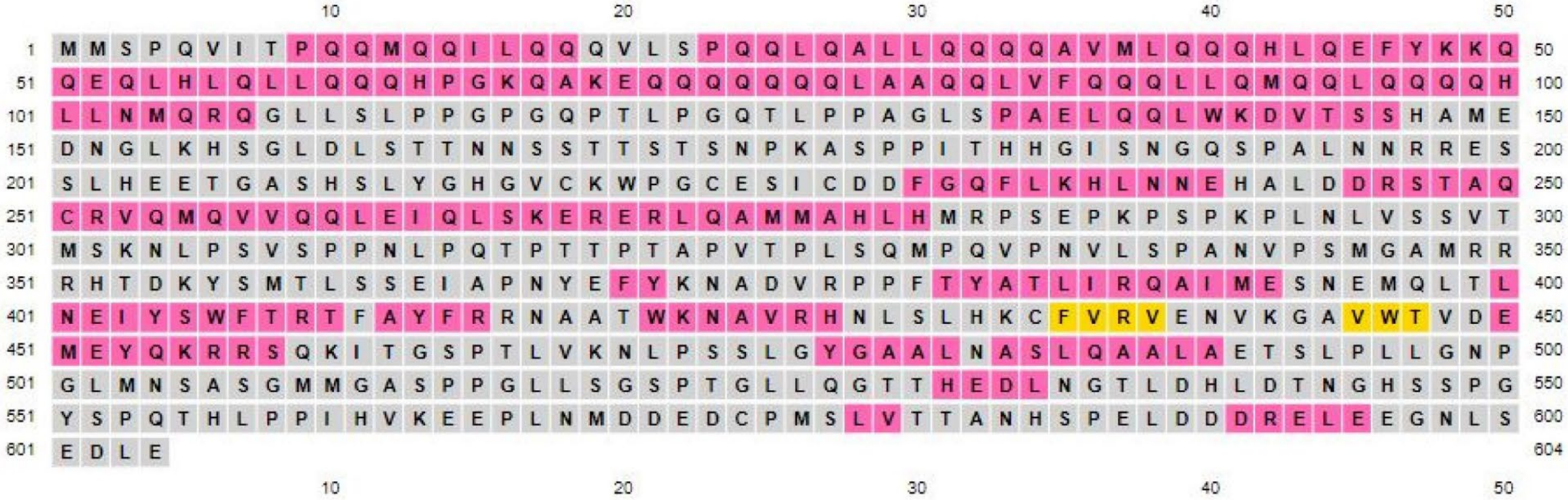

FOXP3

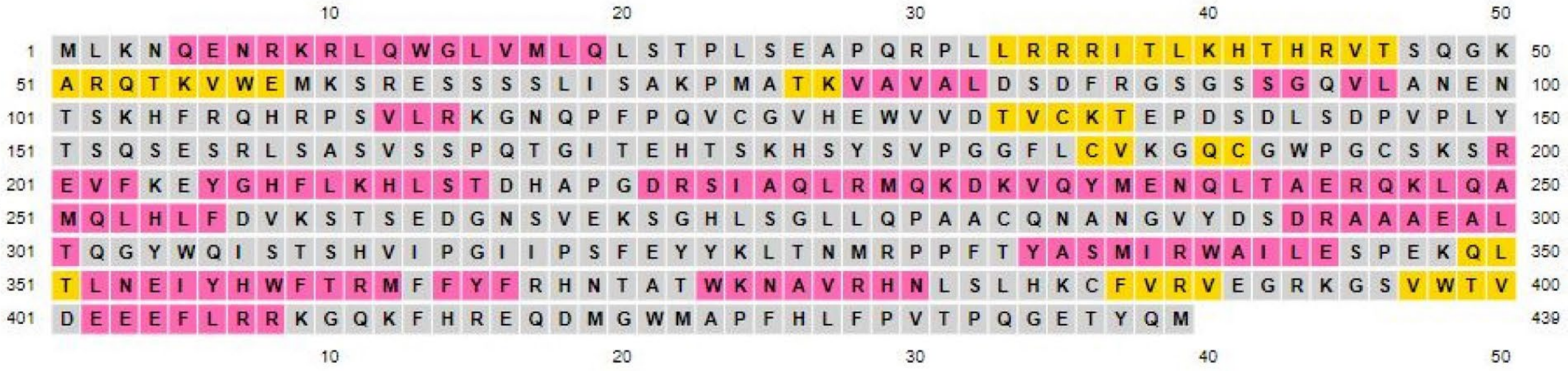
10 20 30 40 50
1 MLKNQENRKRLQWGLVMLQLSTPLSEAPQRPLLRRRITLKHTHRVTSQGK 50
51 ARQTKVWEMKSRESSSSLISAKPMATKVAVALDSDFRGSGSSGQVLANEN 100
101 TSKHFRQHRPSVLRKGNQPFPQVCGVHEWVVDTVCKTEPDSDLSDPVPLY 150
151 TSQSESRLSASVSSPQTGITEHTSKHSYSVPGGFLCVKGQCGWPGCSKSR 200
201 EVFKEYGHFLKHLSTDHAPGDRSIAQLRMQKDKVQYMENQLTAERQKLQA 250
251 MQLHLFDVKSTSEDGNSVEKSGHLSGLLQPAACQNANGVYDSDRAAAEAL 300
301 TQGYWQISTSHVIPGIIPSFEYYKLTNMRPPFTYASMIRWAILESPEKQL 350
351 TLNEIYHWFTRMFFYFRHNTATWKNAVRHNLSLHKCFVRVEGRKGSVWTV 400
401 DEEEFLRRKGQKFHREQDMGWMAPFHLFPVTPQGETYQM 439
10 20 30 40 50
