# Supplementary material for: Investigating the role of FOX gene family in development and stress response in Labeo rohita: A multi-faceted analysis of phylogeny and genome characterization
Source: PLoS One. 2025 Aug 21;20(8):e0323740. doi: 10.1371/journal.pone.0323740 (PMC12370200; doi:10.1371/journal.pone.0323740)
Supplement: S2 Fig — (PDF) [file pone.0323740.s003.pdf]

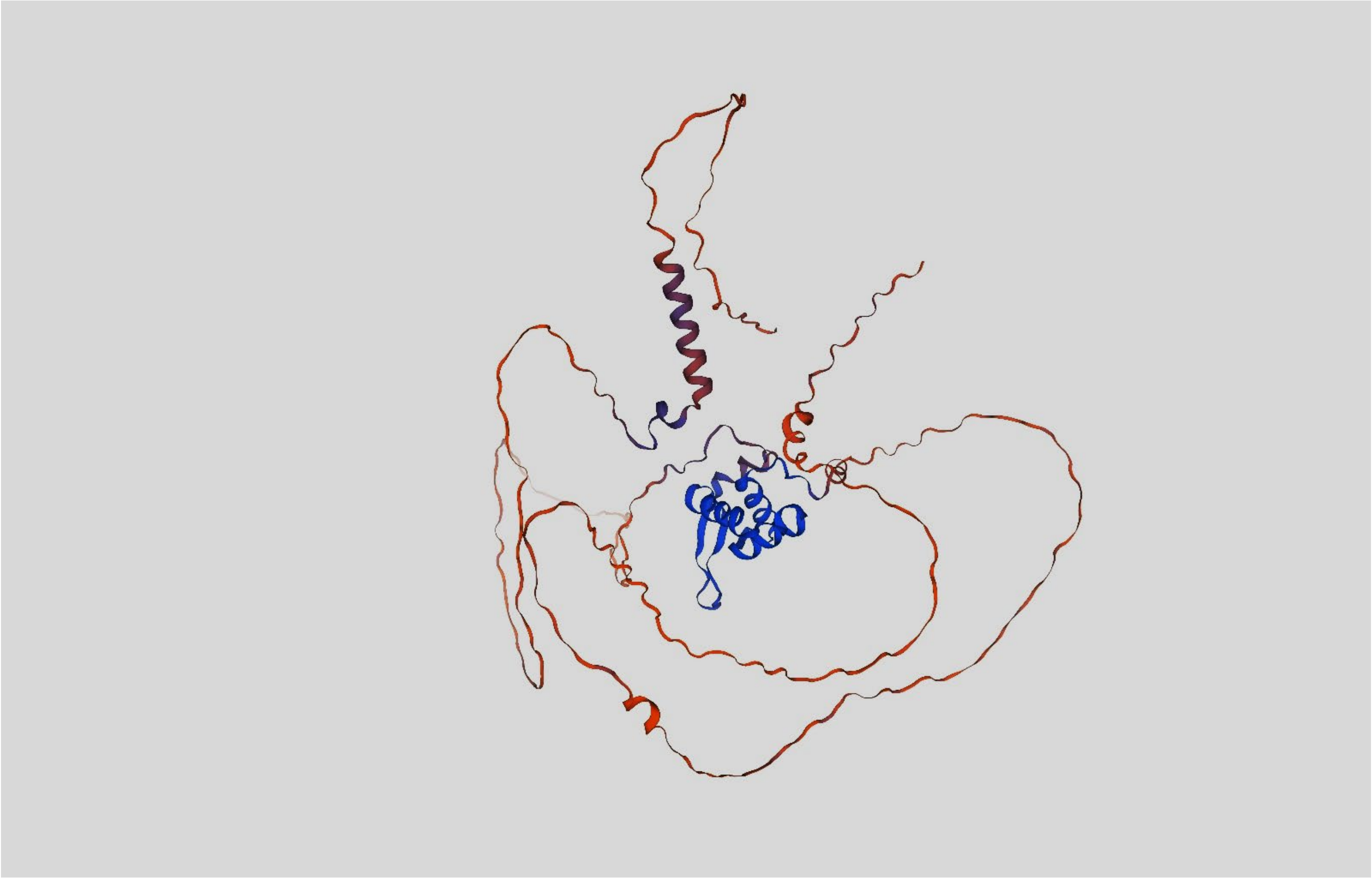

FOXAI

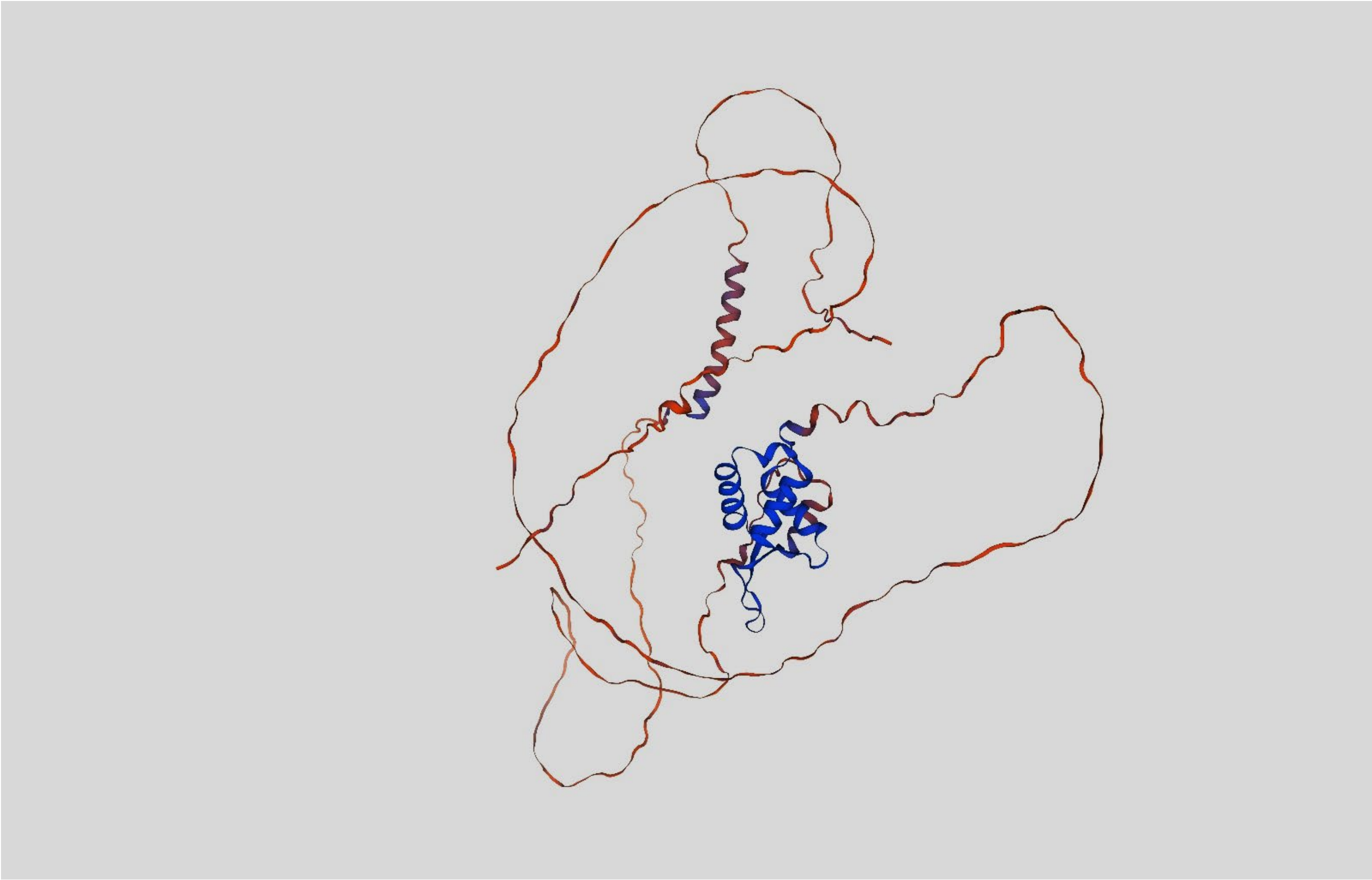

FOXA2

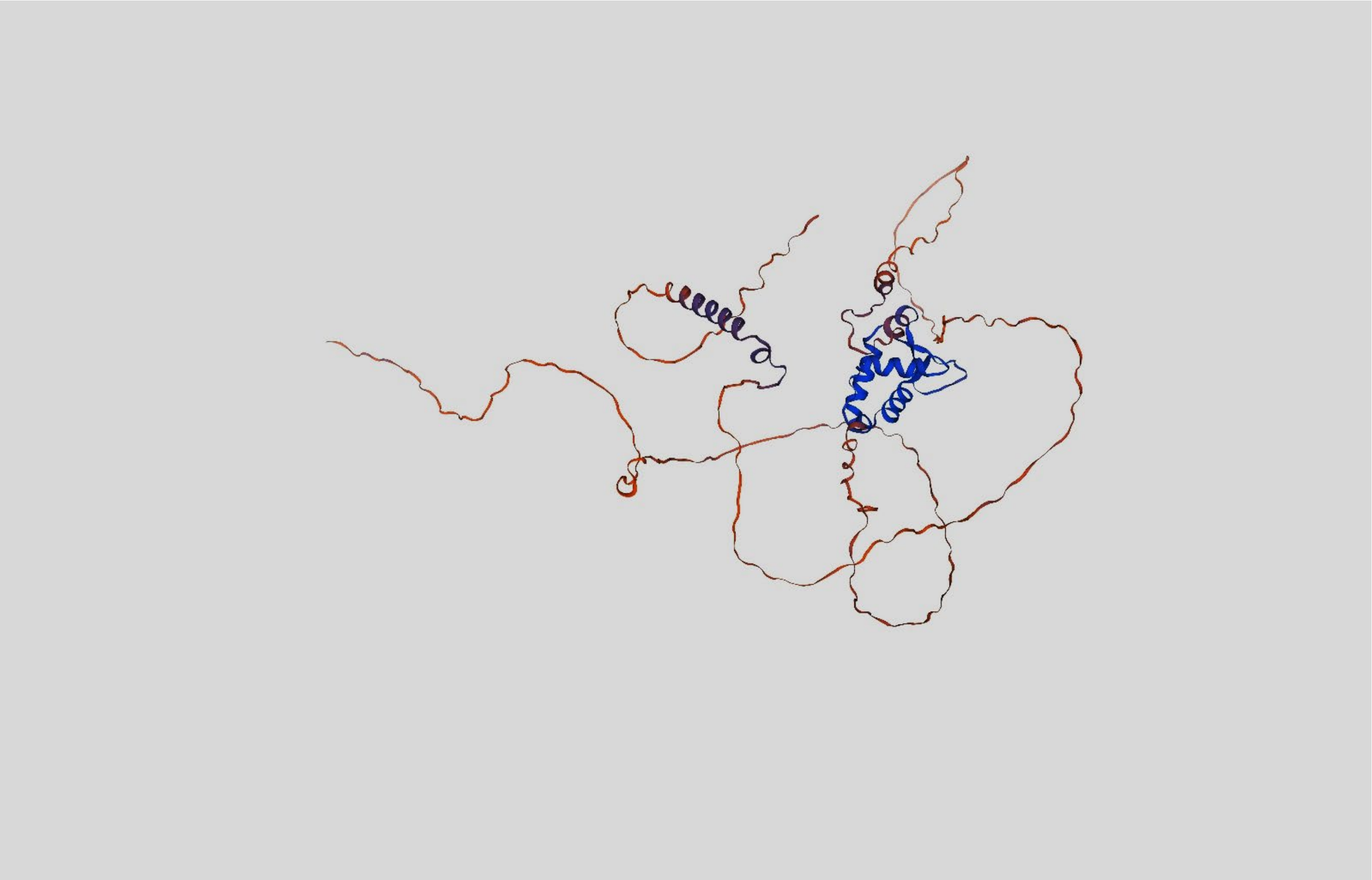

FOXA3

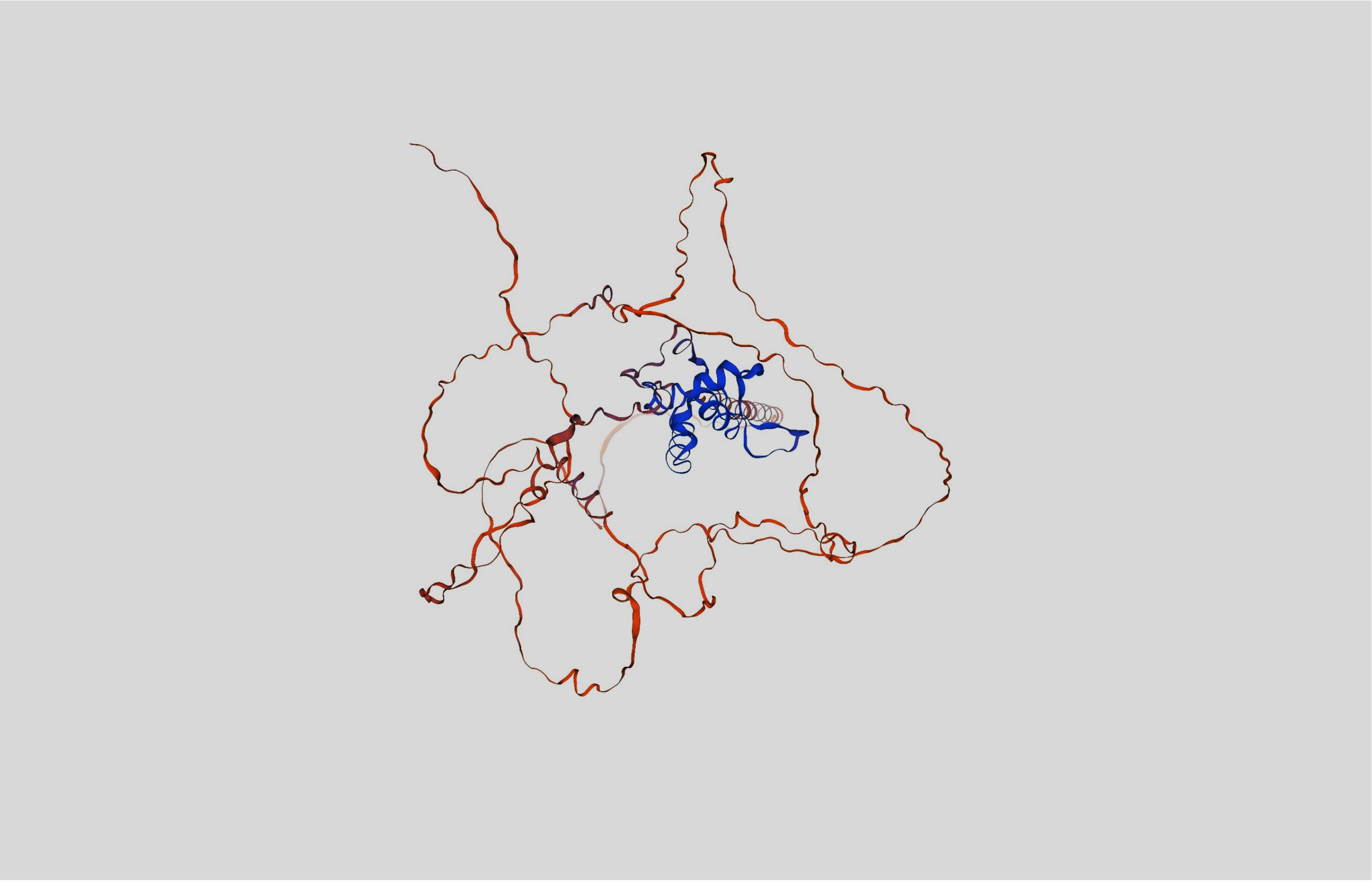

FOXC1

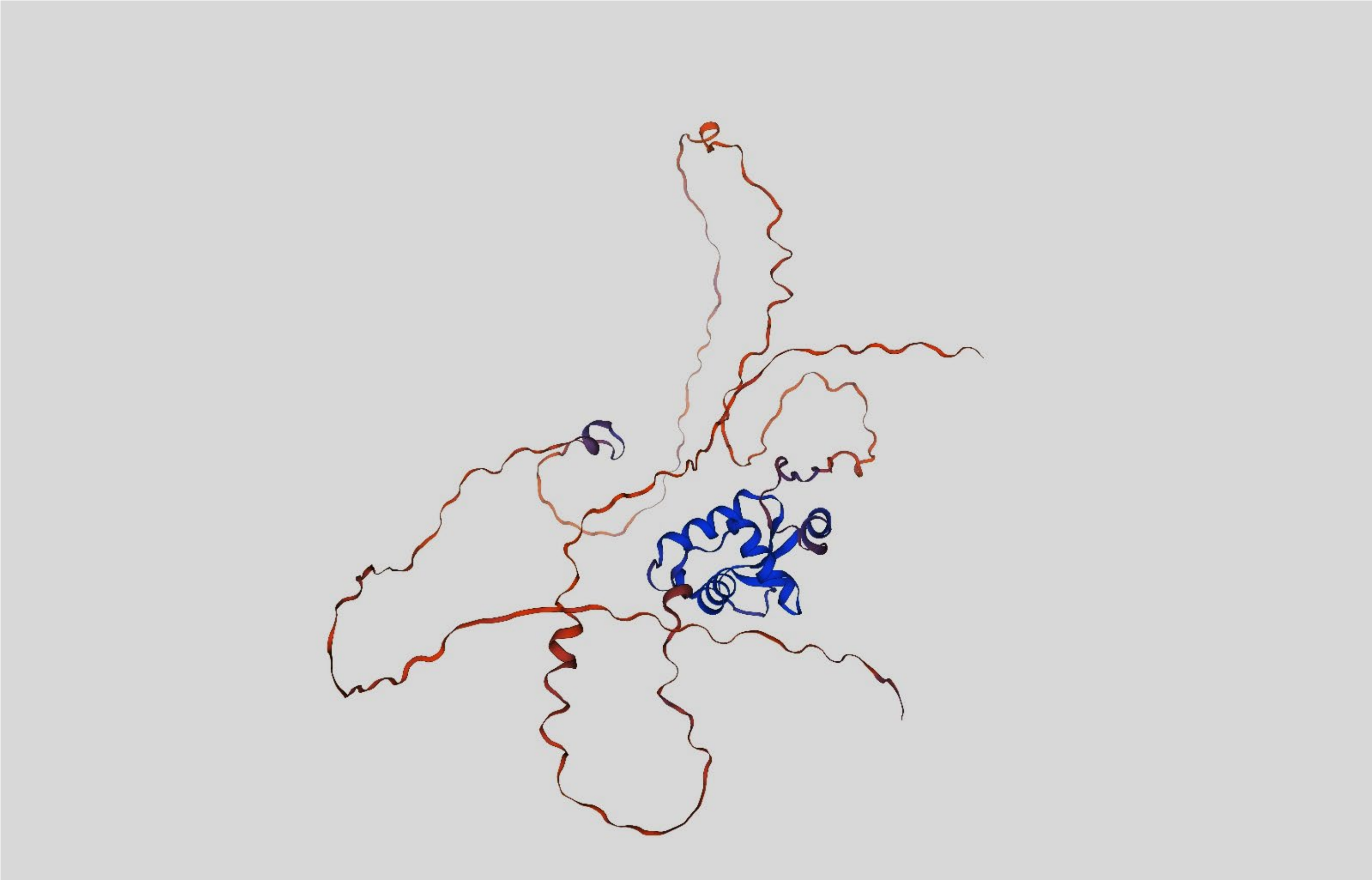

FOXD1

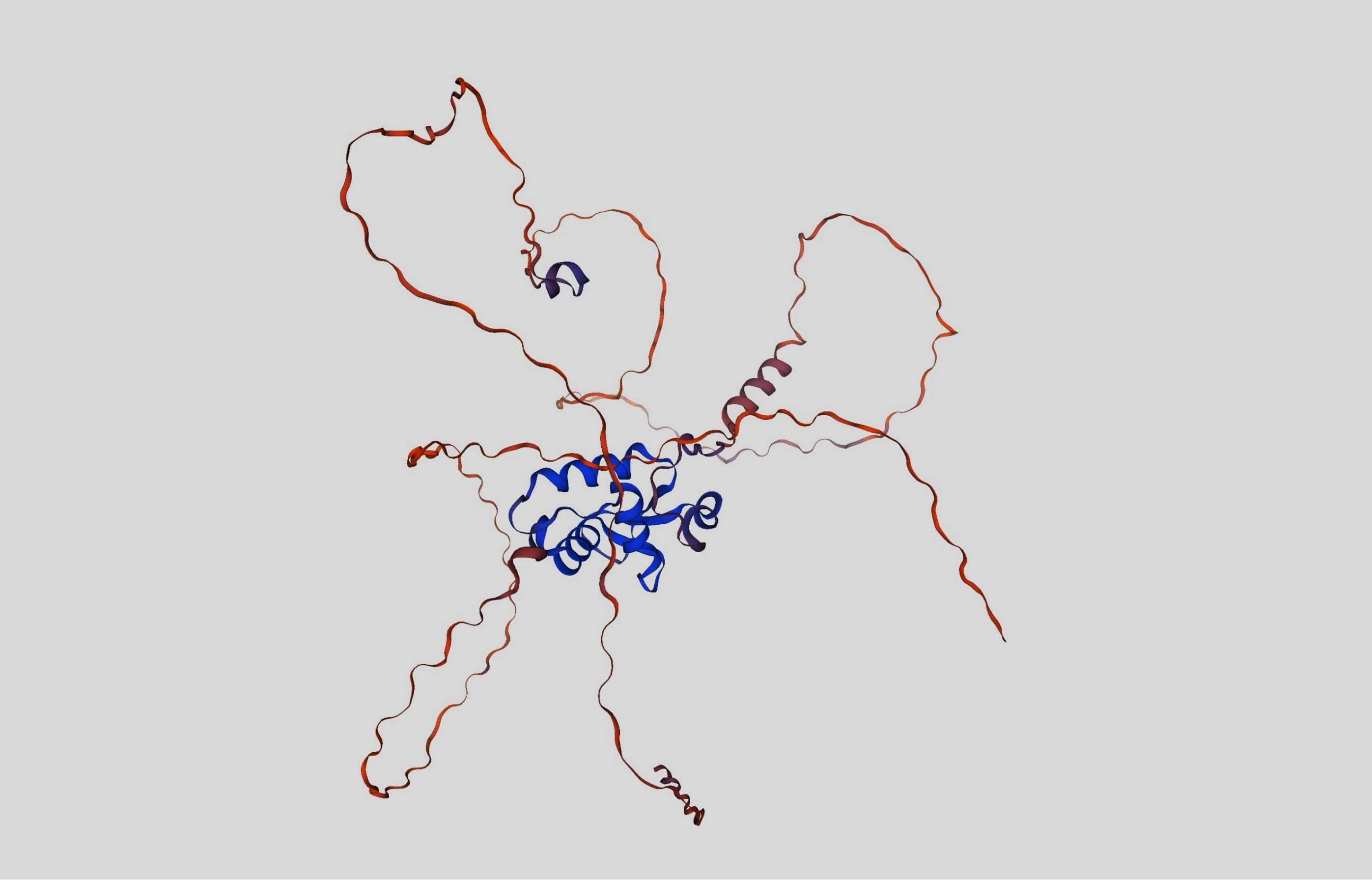

FOXD3

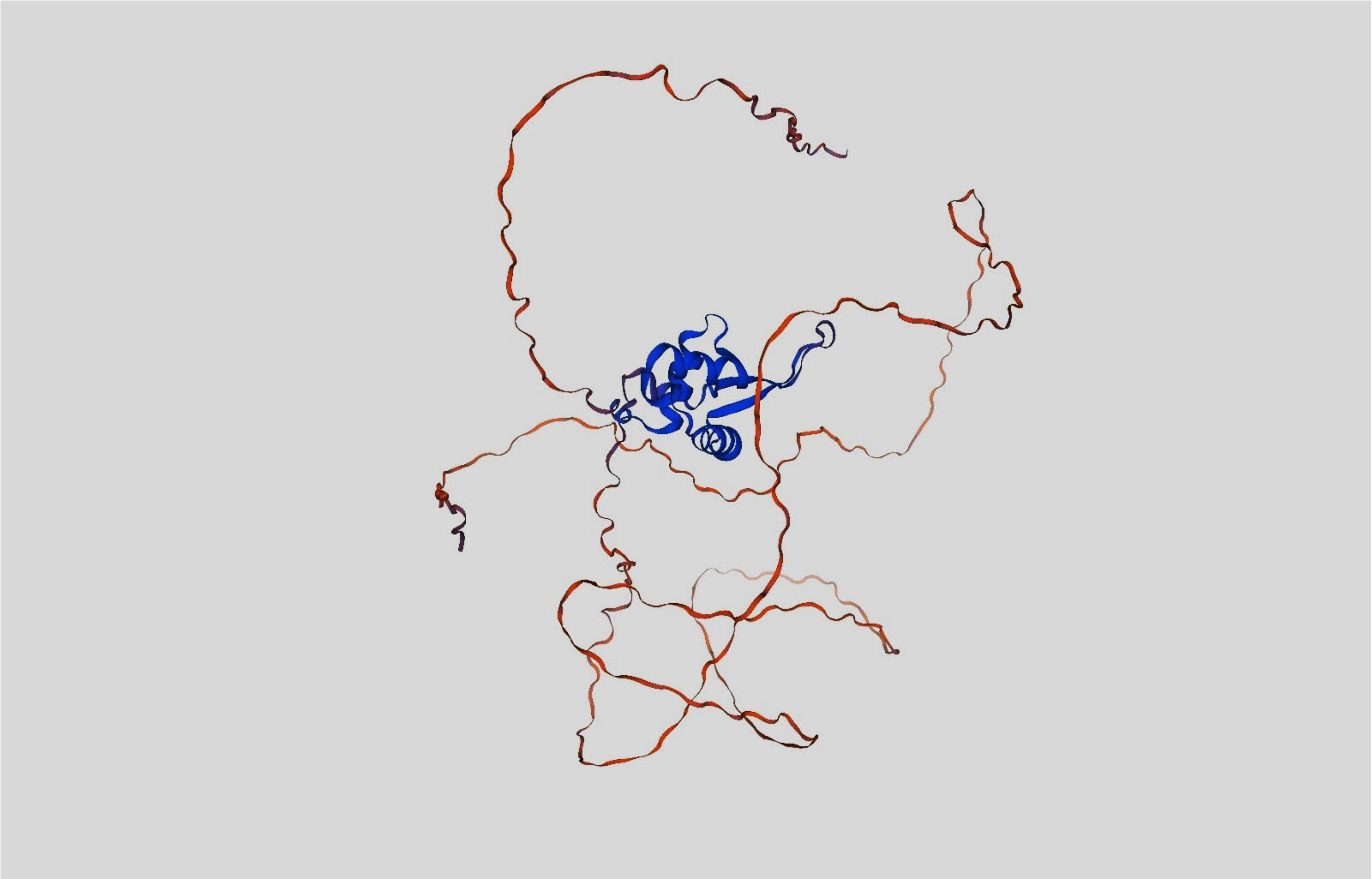

FOXF1

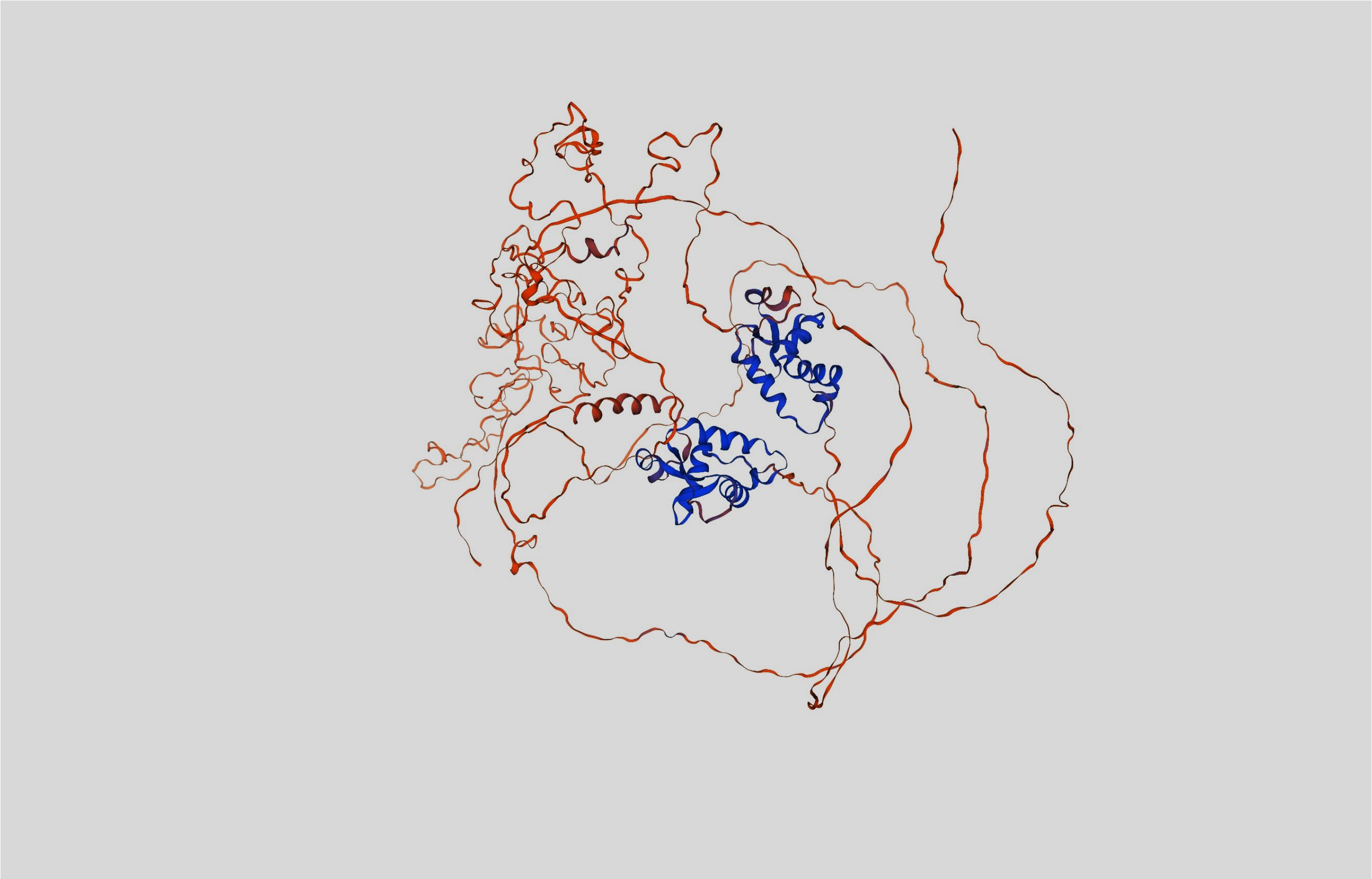

FOXF2

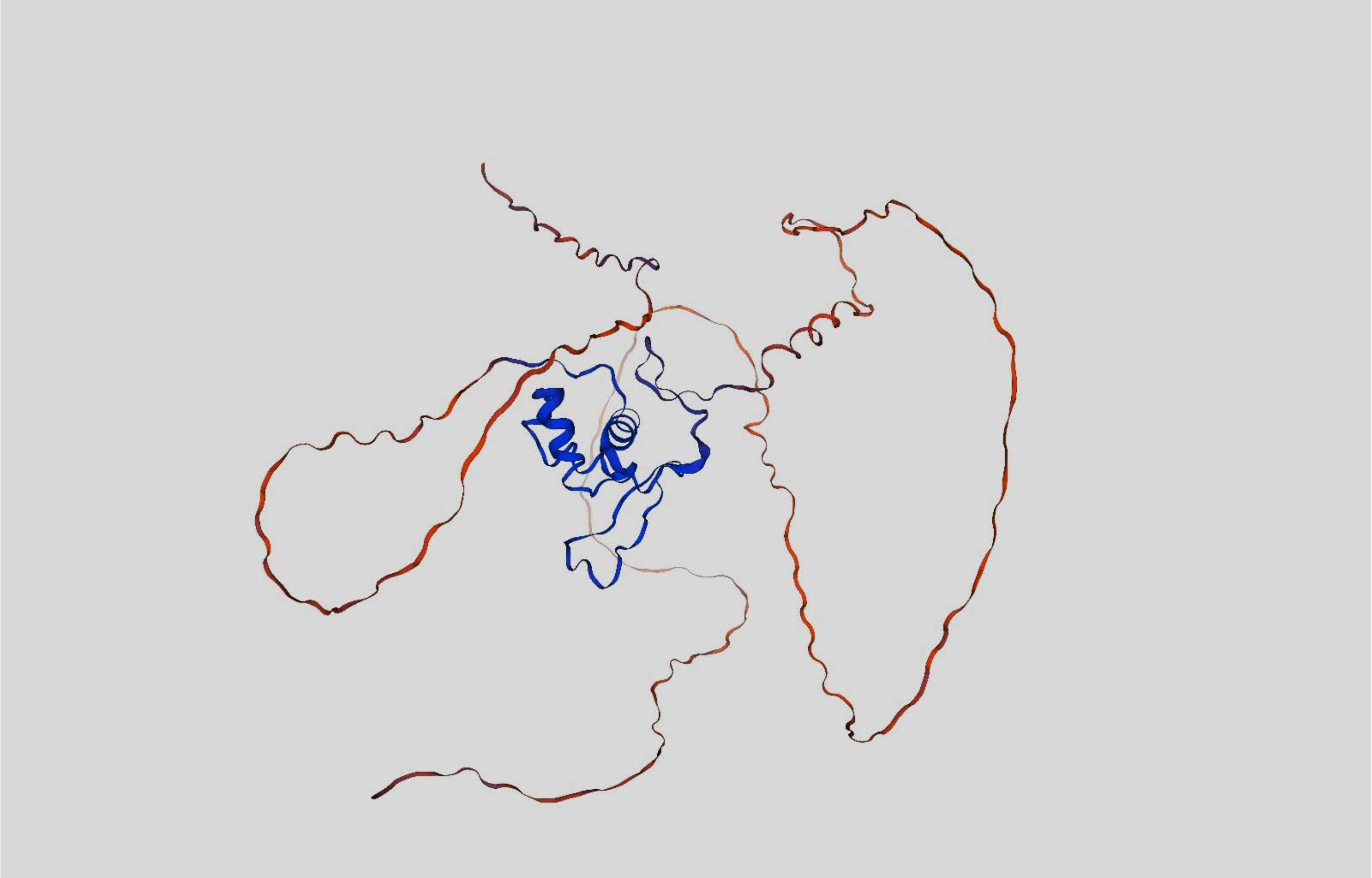

FOXG1

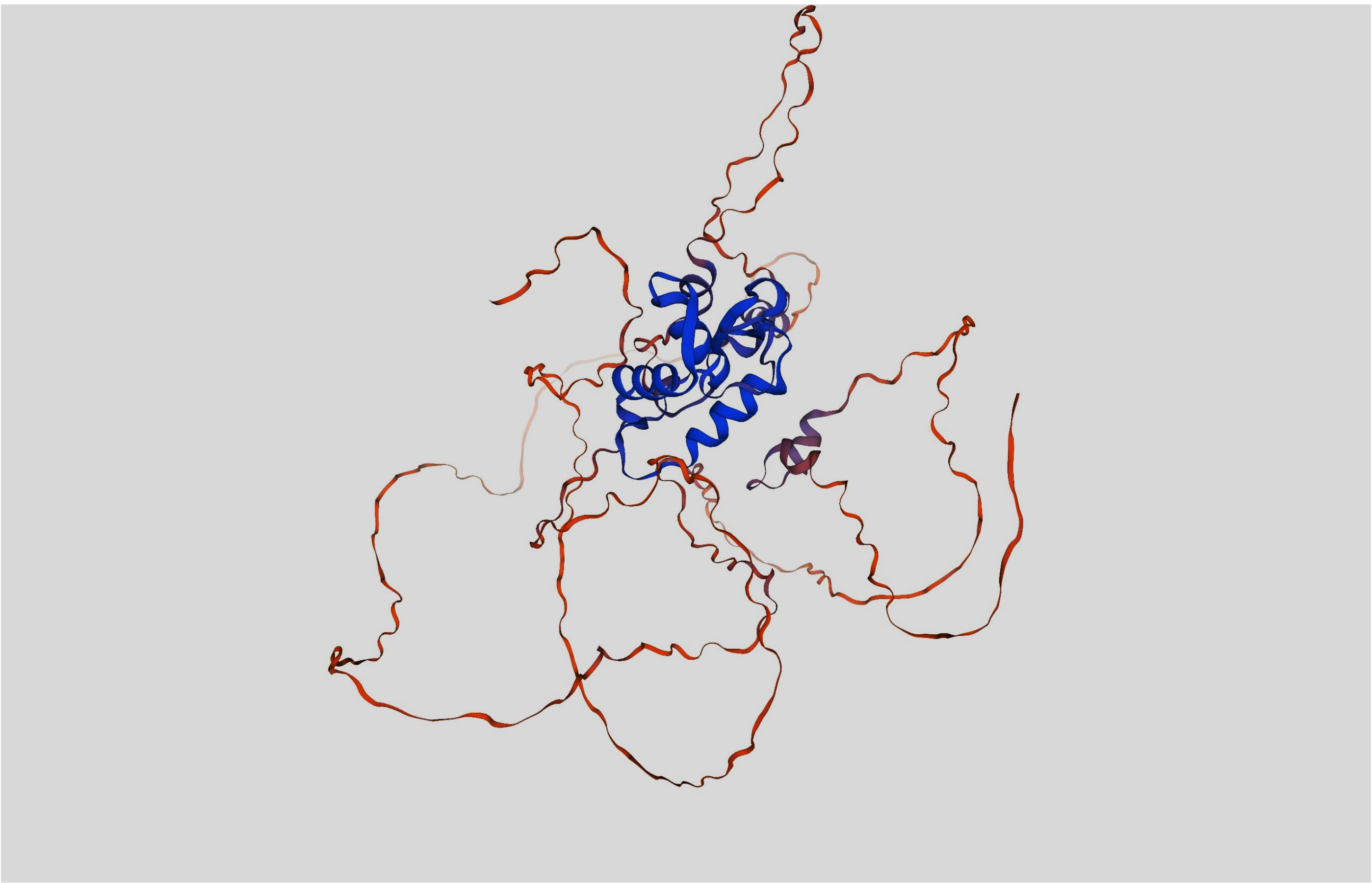

FOXH1

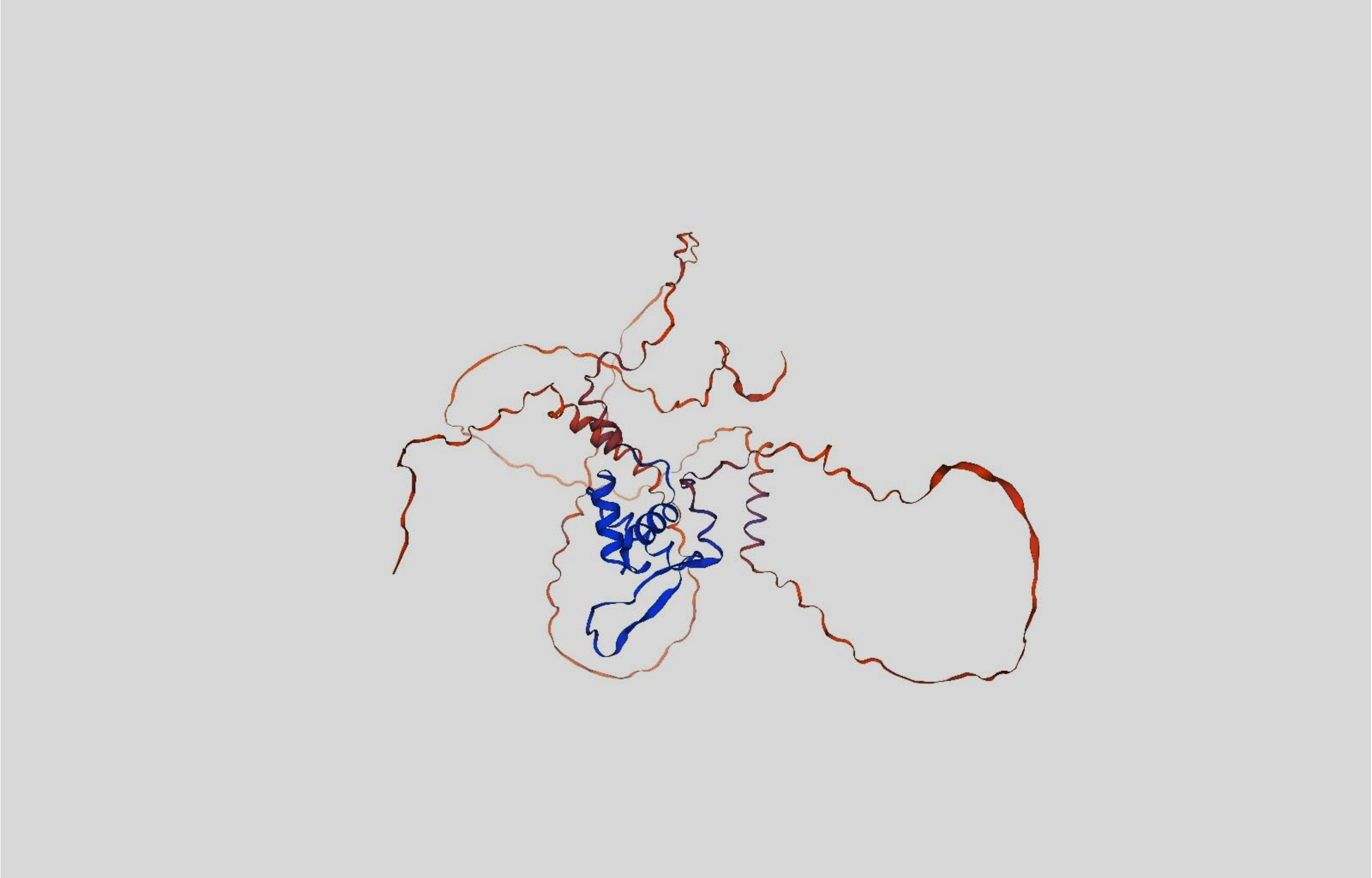

FOXI1

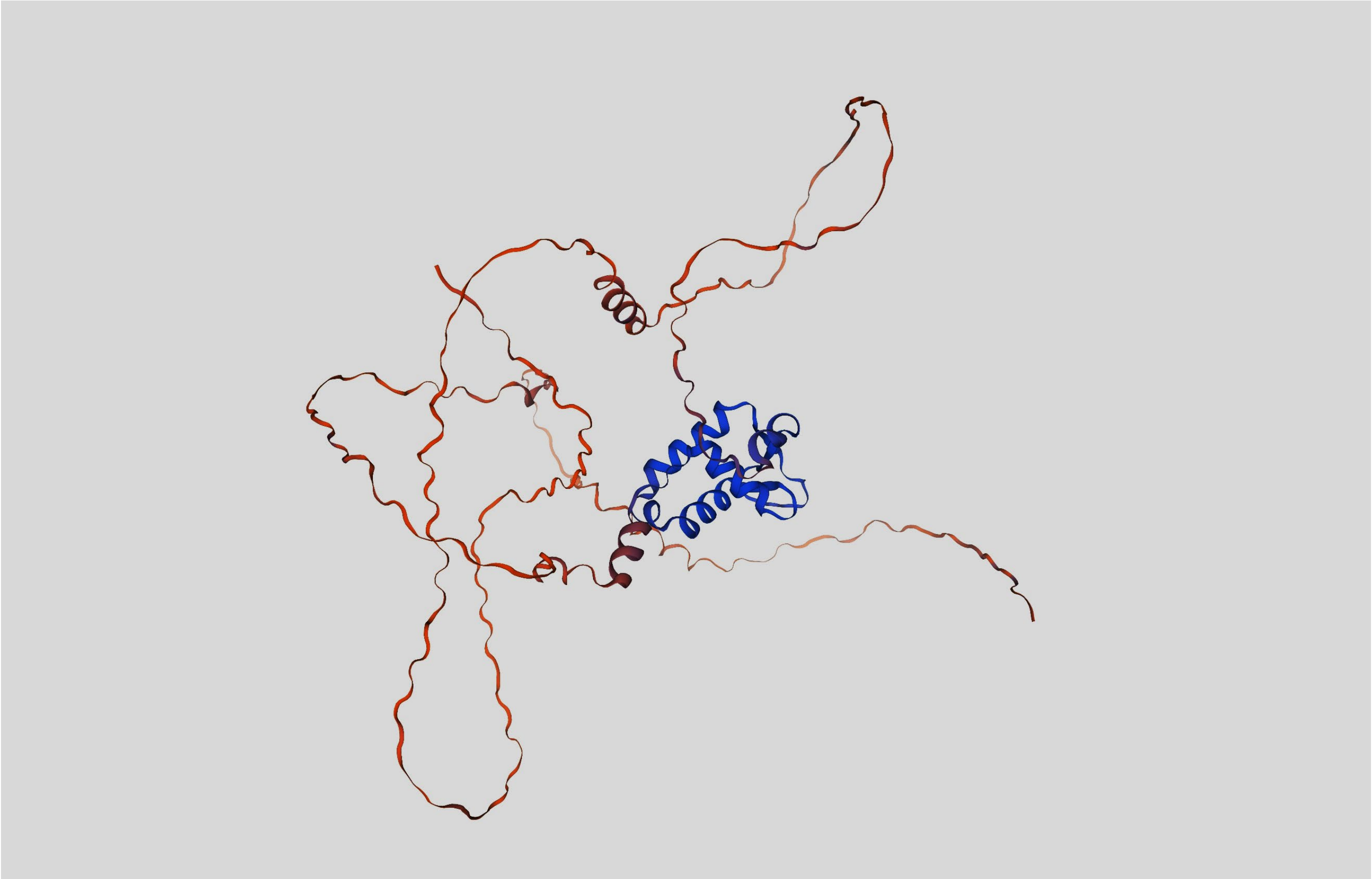

FOXI2

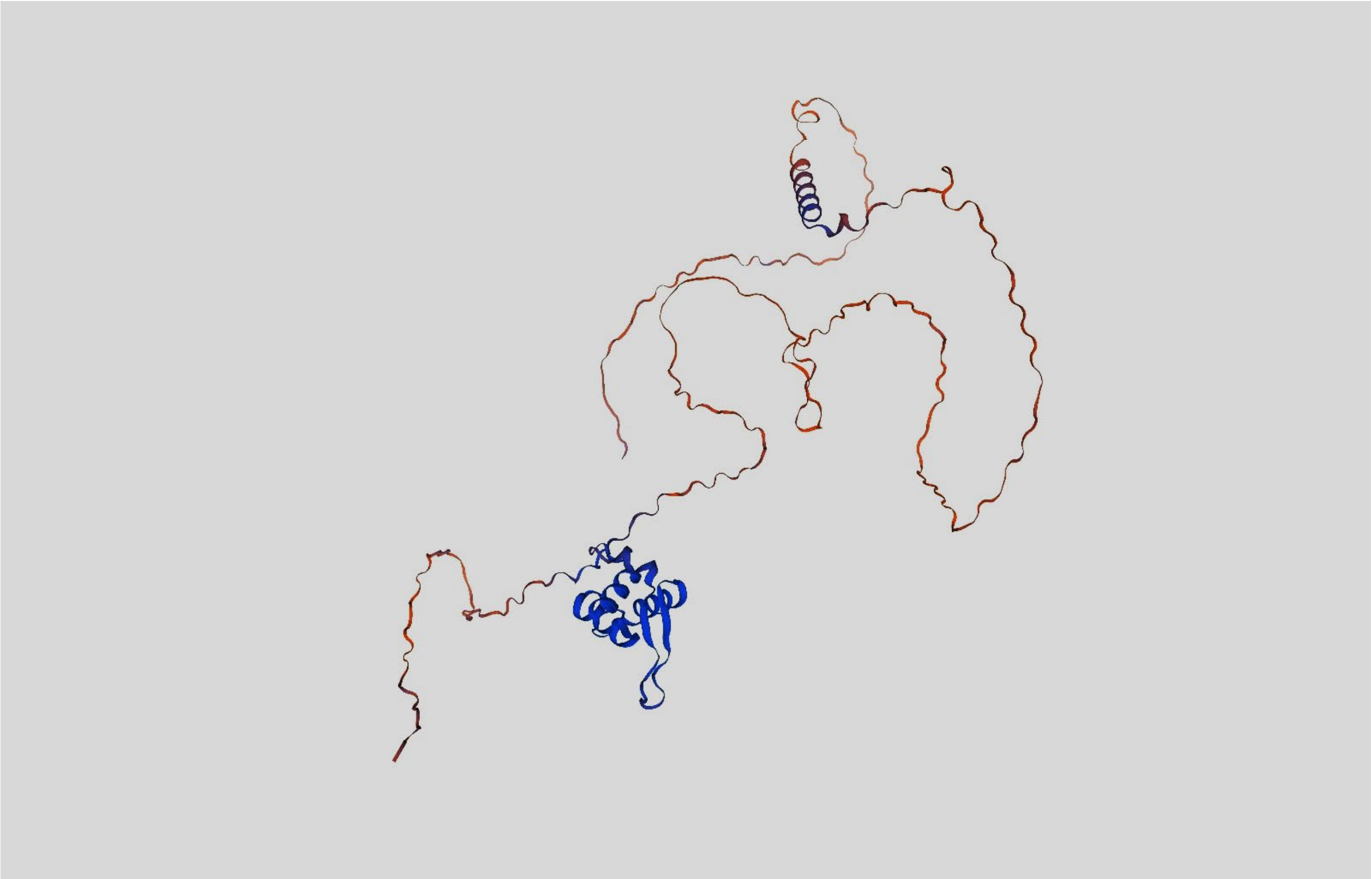

FOXL1

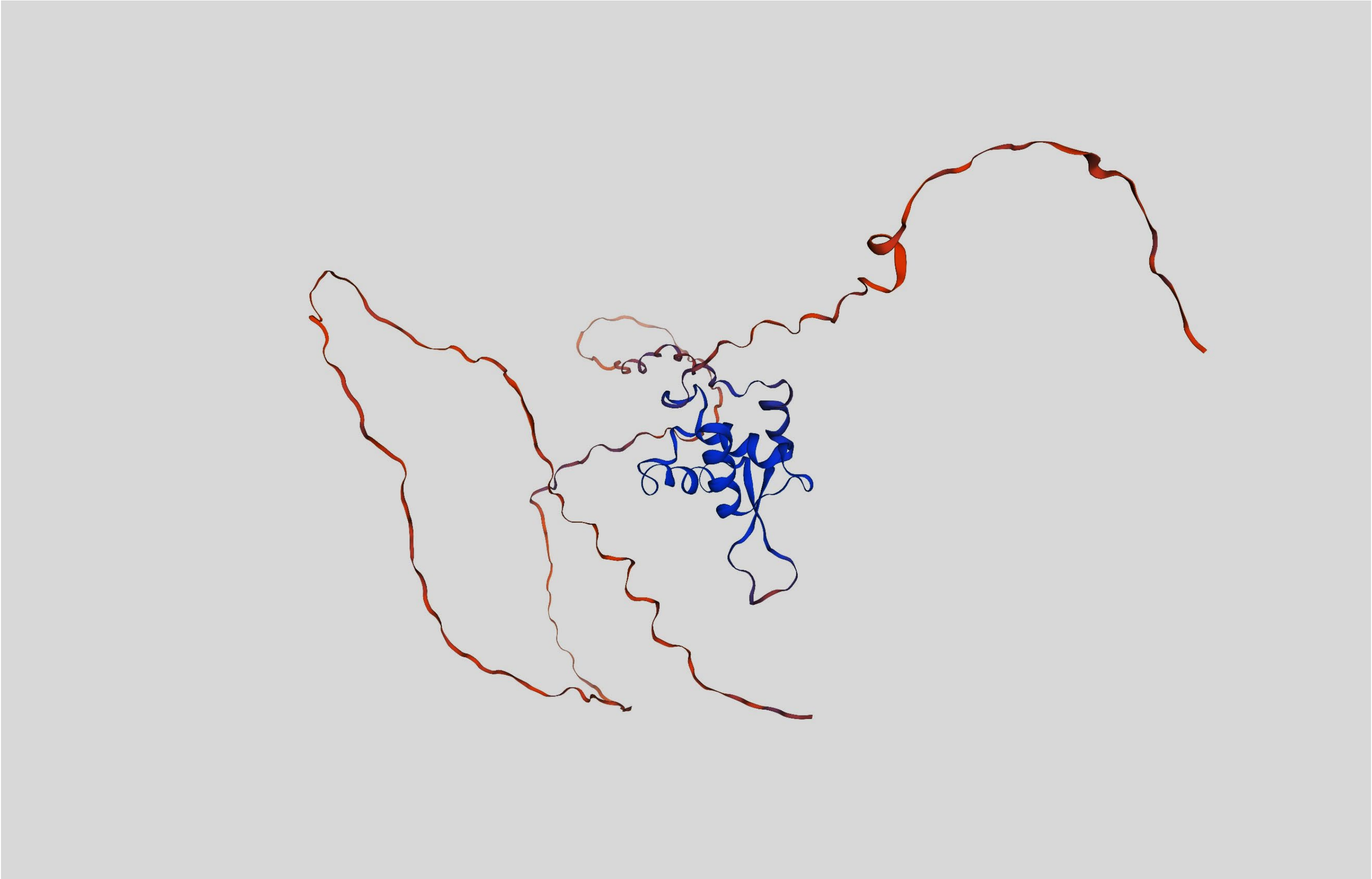

FOXL2

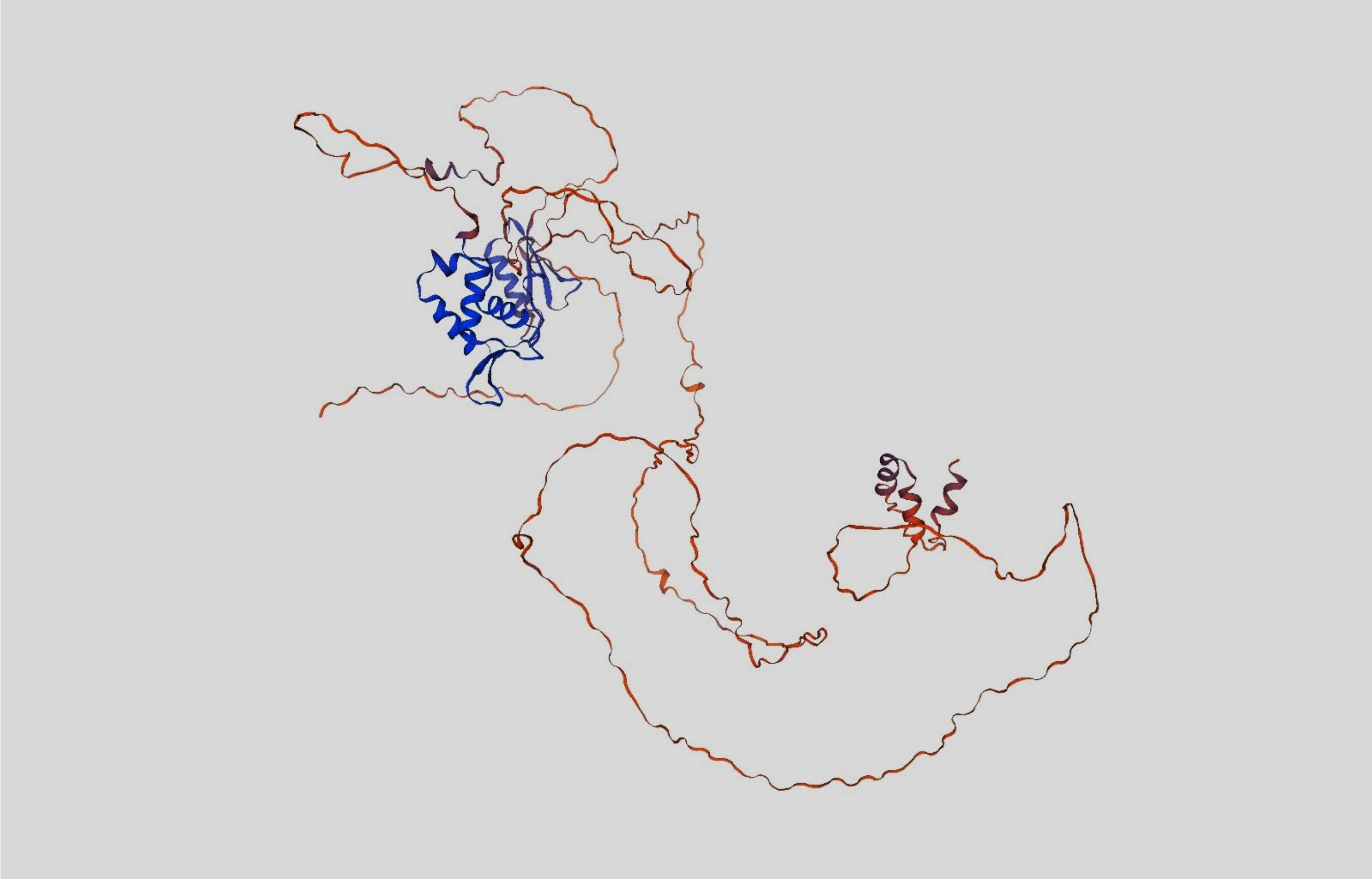

FOXM1

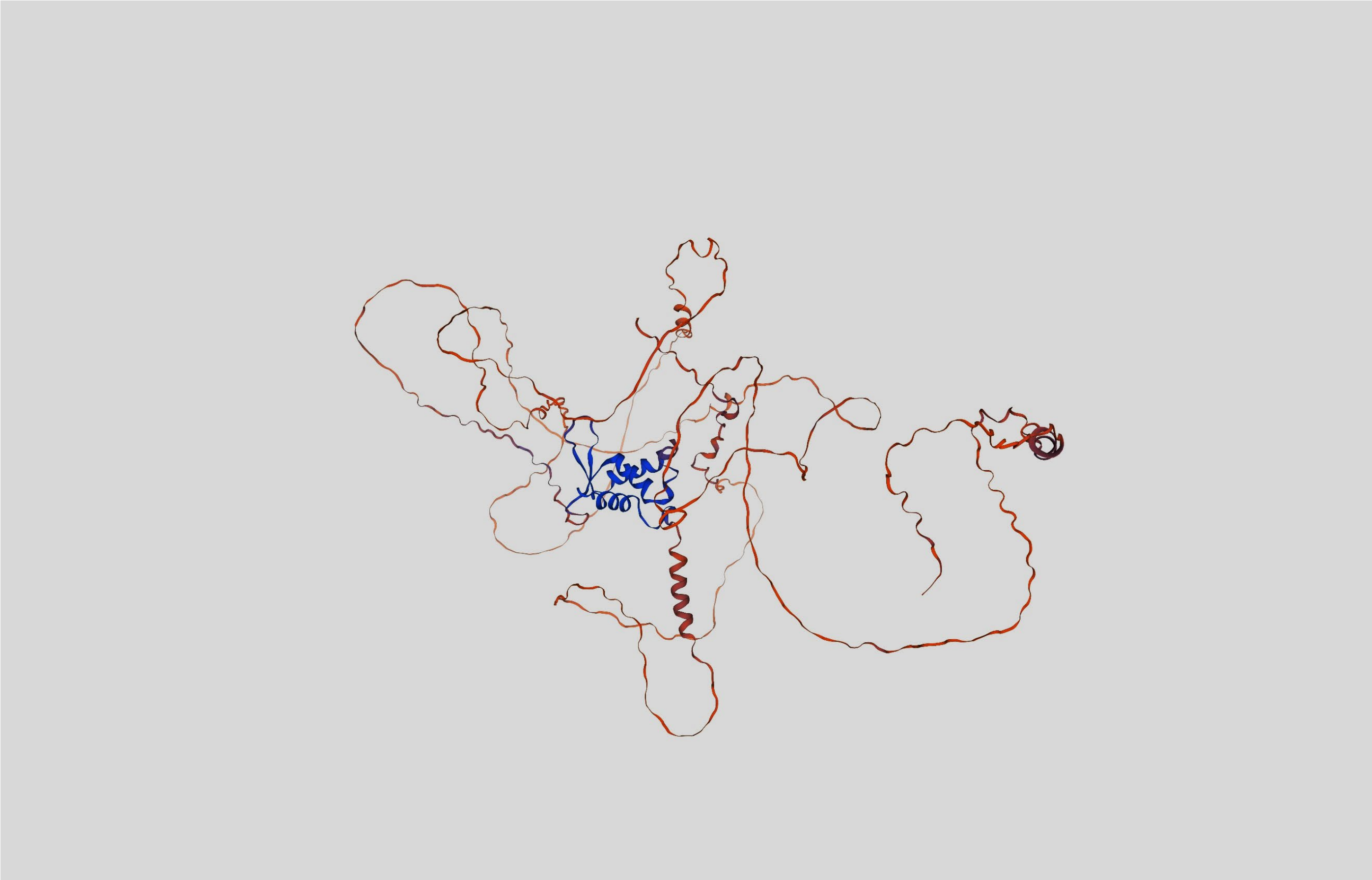

FOXO1

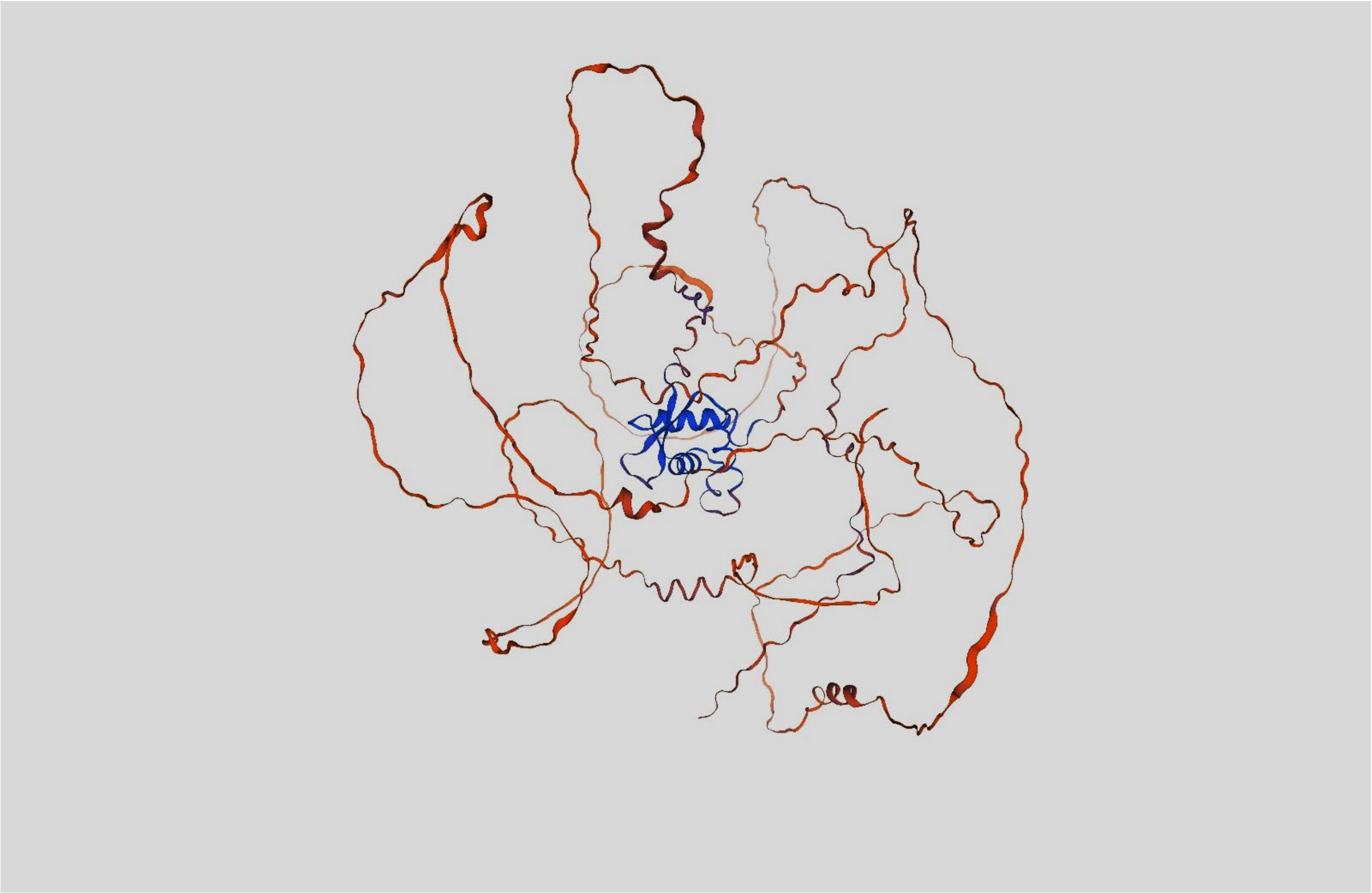

v

FOXO3

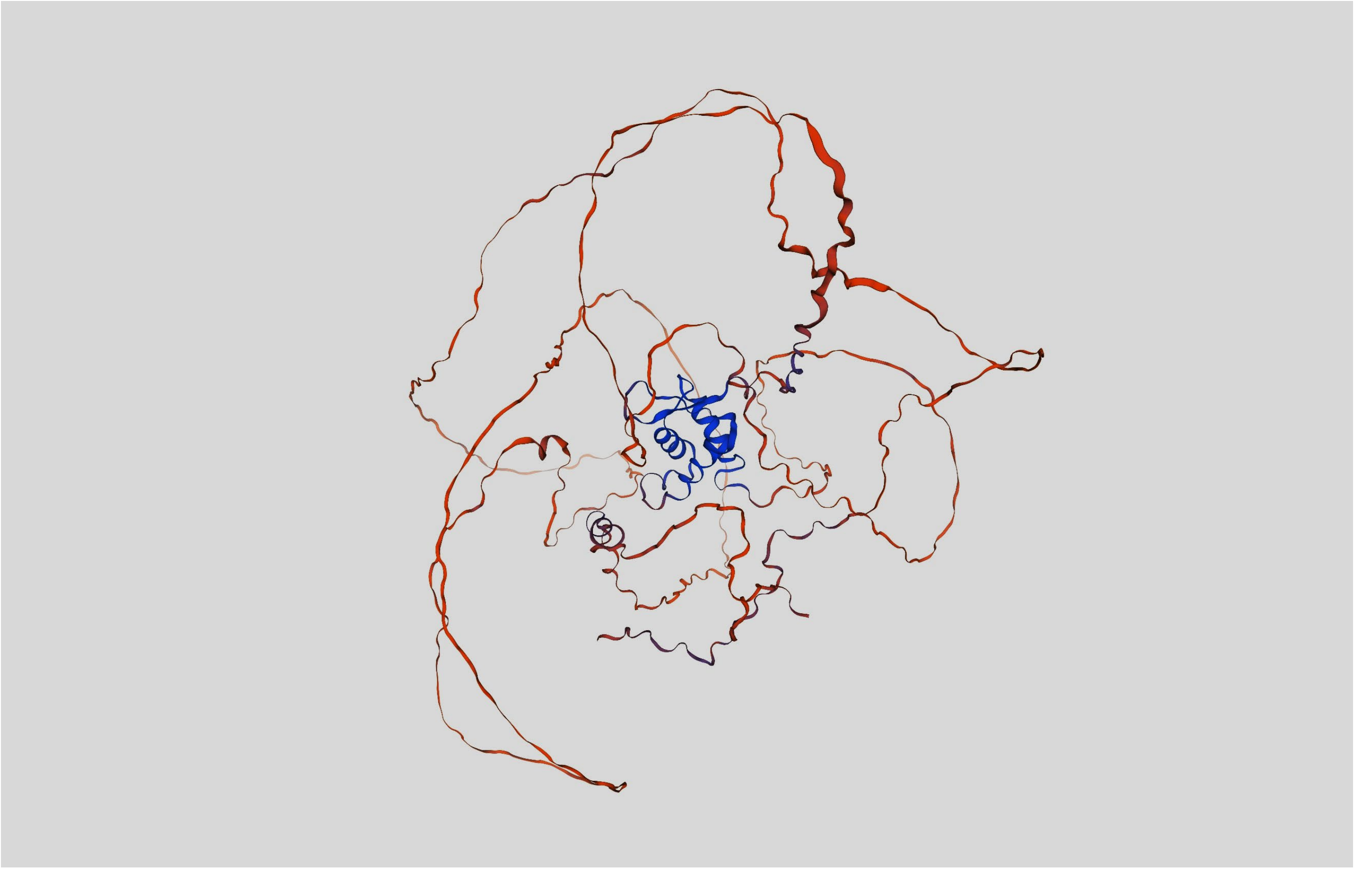

FOXO4

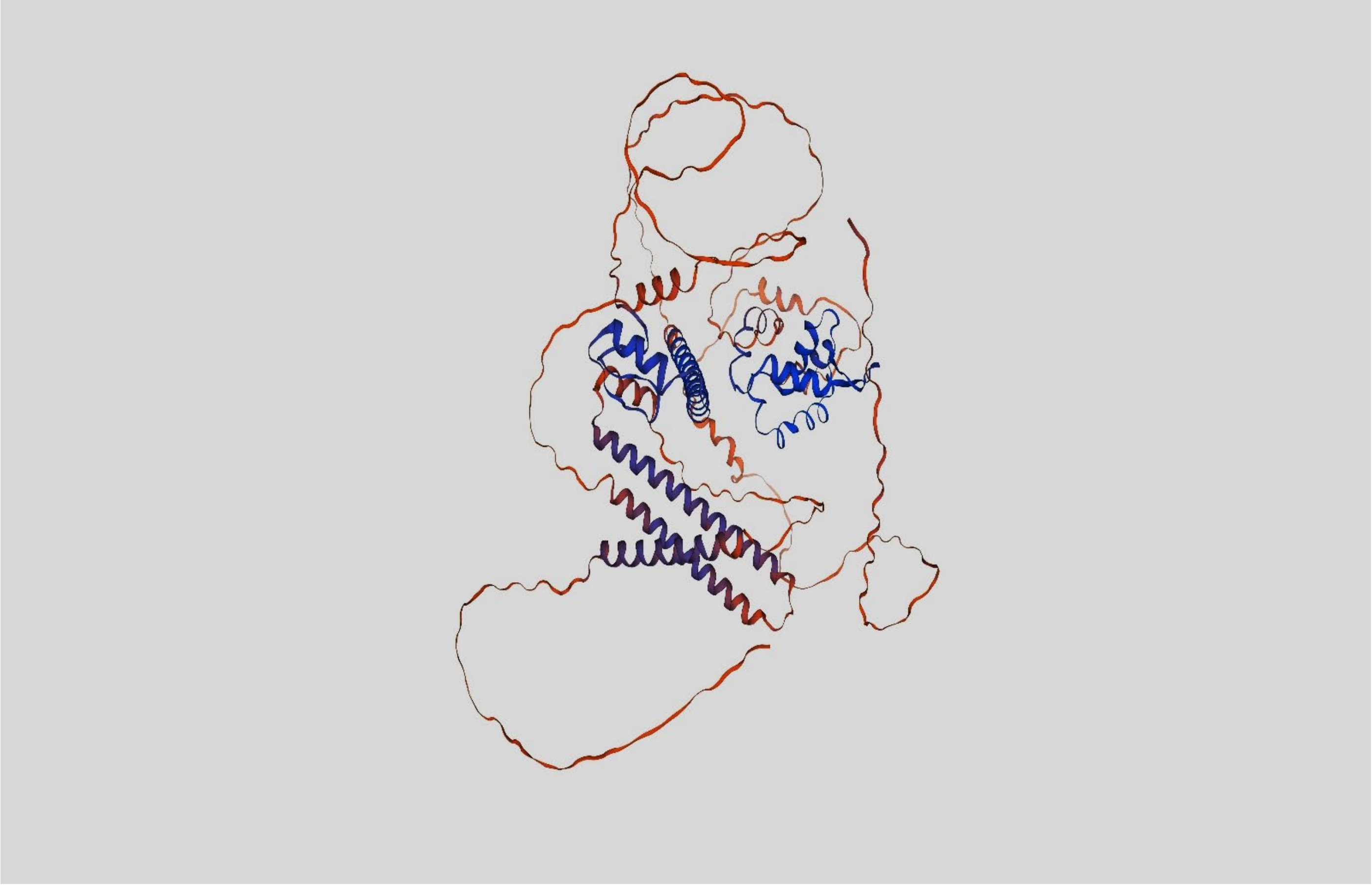

FOXP1

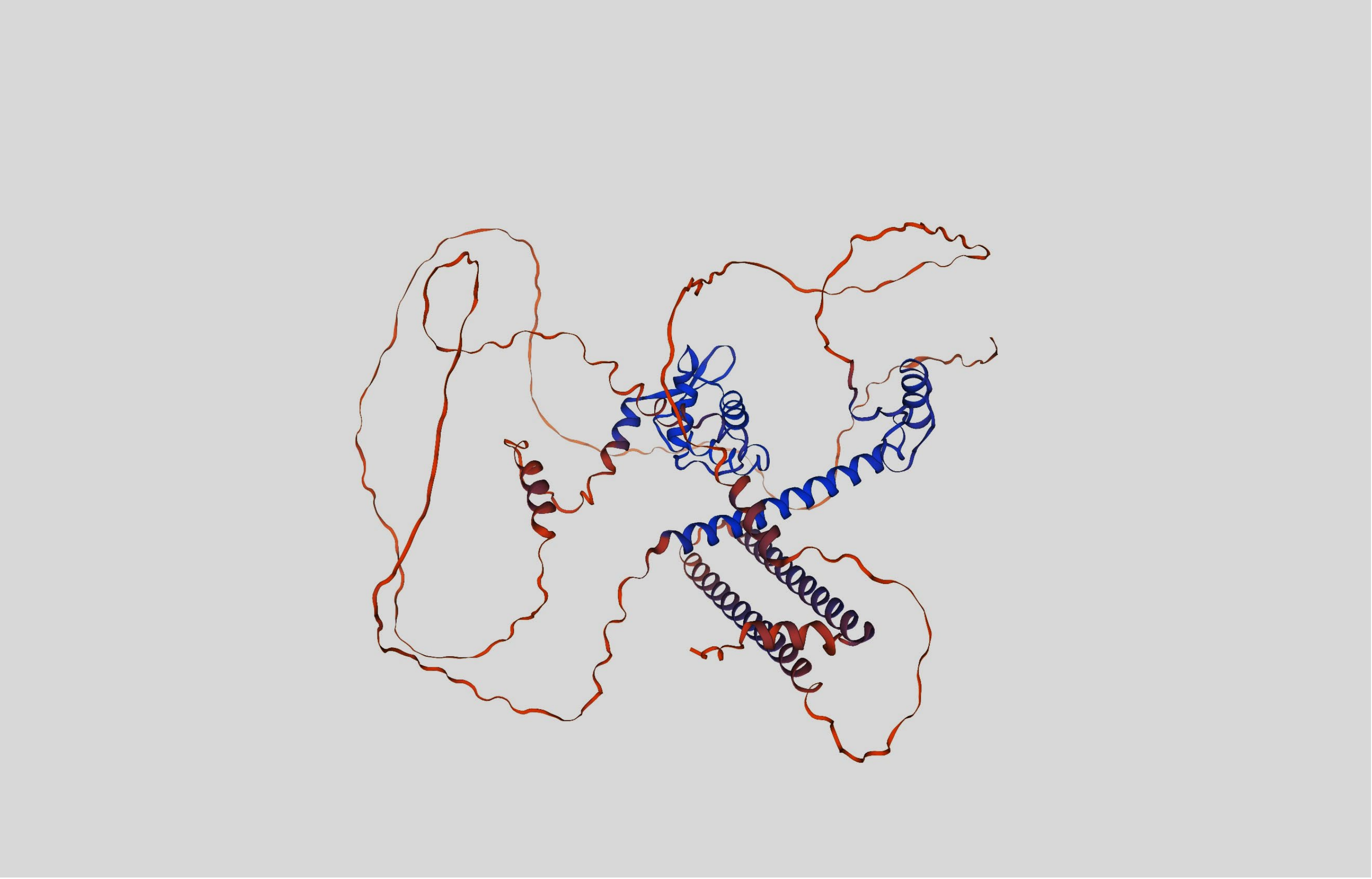

FOXP2

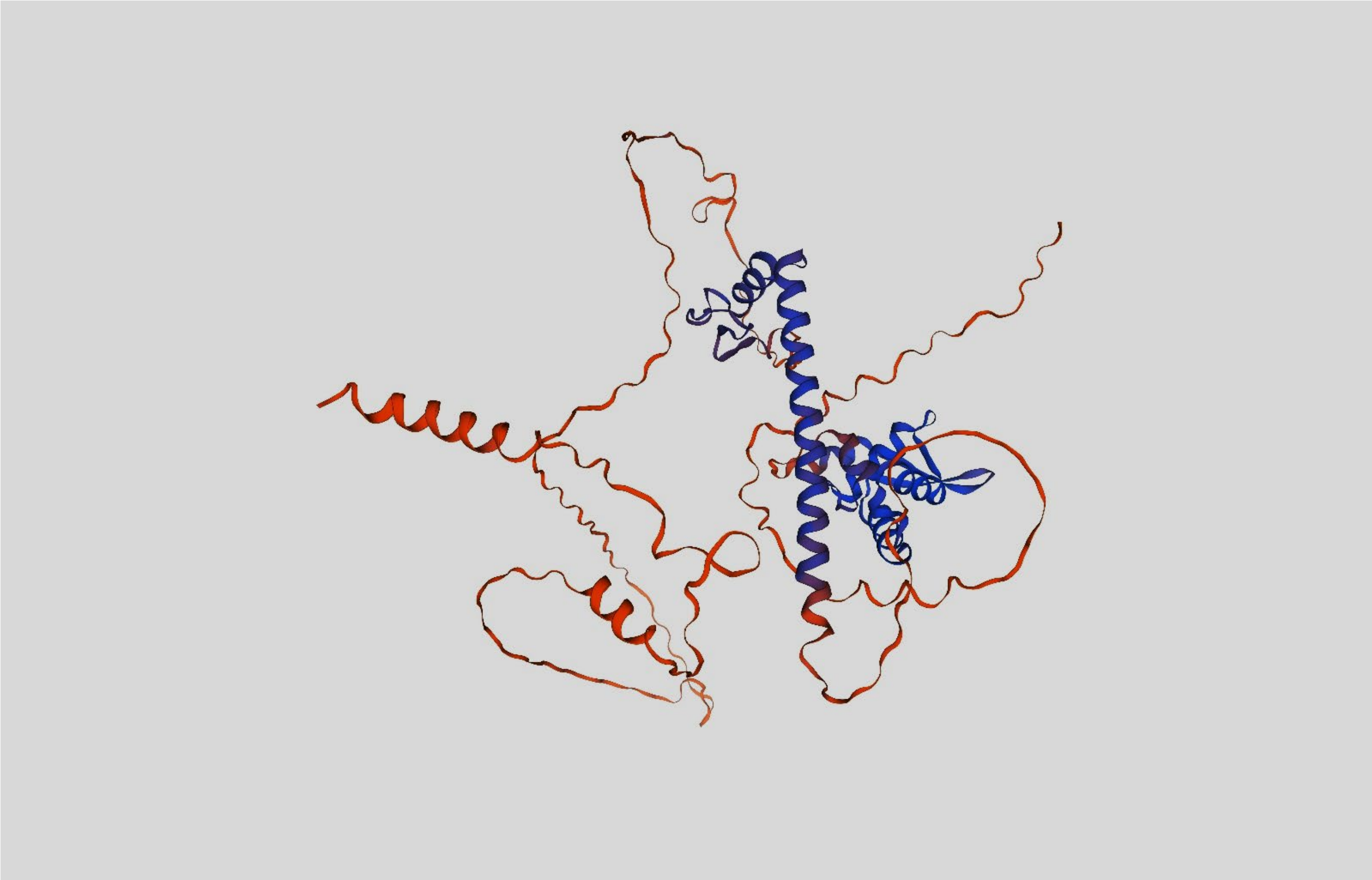

FOXP3
