# Supplementary material for: Investigating the role of FOX gene family in development and stress response in Labeo rohita: A multi-faceted analysis of phylogeny and genome characterization
Source: PLoS One. 2025 Aug 21;20(8):e0323740. doi: 10.1371/journal.pone.0323740 (PMC12370200; doi:10.1371/journal.pone.0323740)
Supplement: Attachment — Submitted filename: Response to Reviewers.docx [file pone.0323740.s004.docx]

Dear Editor and Reviewers,

We would like to express our sincere gratitude for the time and effort invested in reviewing our manuscript entitled “**Investigating the Role of FOX Gene Family in Development and Stress Response in *Labeo rohita*: A Multi-faceted Analysis of Phylogeny and Genome Characterization**.” Your insightful comments have been invaluable in enhancing the quality and clarity of our work. Below, we provide a detailed, point-by-point response to all comments raised.

**Response to Reviewer #1**

**Comment:** “The Abstract section is not justified.”

**Response:** We have revised the abstract to ensure full justification in accordance with the journal’s formatting guidelines. The updated abstract now meets the required presentation style.

**Comment:** “If possible, for secondary structure, authors are advised to use more than one program for their validation with suitable references.”

**Response:** We thank the reviewer for the suggestion. Our manuscript now specifies that FOX protein secondary structure was predicted using PSIPRED and independently validated with Pyre2, with both methods yielding consistent results. All relevant references are now provided in the manuscript.

**Comment:** “For using iTol, the file type used for designing the phylogeny should be mentioned.”

**Response:** We have now specified in the Methods section that the phylogenetic tree was generated using the Newick format file generated by MEGA11.

**Comment:** “The study only highlights the available sequences of the FOX gene in the NCBI database. The authors haven’t confirmed the 5’-UTR and 3’-Poly A tail by using RACE PCR. Using the terms in conclusion, like detailed genome-wide analysis, might not be true. If possible, avoid using terms like that.”

**Response:** We acknowledge this concern and have revised the manuscript accordingly. The text now clearly states that the study is based on computational analysis of sequences available in the NCBI database. The term “detailed genome-wide analysis” has been replaced with “comprehensive computational analysis” to more accurately reflect the scope of our work.

**Comment:** “The discussion section is well written. The authors advised to revise accordingly.”

**Response:** We appreciate the positive feedback. Minor revisions have been made in the discussion to enhance clarity and to improve the integration and correlation of the results.

**Response to Reviewer #2**

**Comment:** “In Abstract, authors should specifically state the need of this research.”

**Response:** The abstract has been updated to explicitly state the motivation behind our study, highlighting the significance of understanding the role of FOX genes in the developmental and stress response mechanisms of *Labeo rohita*.

**Comment:** “Bootstrap consensus values were calculated for each node in the analysis……………. In Figure 1, no bootstrap values are visible.”

**Response:** We have revised Figure 1 to include bootstrap values for each node. The figure legend has been updated to describe these values and the method of calculation in detail.

**Comment:** “Figure caption should be improved with details.”

**Response:** The caption for Figure 1 has been expanded to include additional details.

**Comment:** “Discussion section should be revised by the authors. Try to correlate the results obtained with each other.”

**Response:** We have restructured the discussion to provide a more cohesive interpretation of our findings. The revised version more clearly correlates the outcomes.

**Comment:** “Authors are advised to use multiple programs for validation with appropriate references.”

**Response:** In our study, FOX protein secondary structure was accurately predicted using PSIPRED and validated with Pyre2 for 2D analysis. We have now incorporated SWISS-MODEL for 3D structure validation, and relevant references have been added to the manuscript.

**Comment:** “4.2. Phylogenetic Analysis of FOX genes superfamily: Authors should write their methodology in details.”

**Response:** We have expanded Section 4.2 to include a detailed description of our phylogenetic analysis methodology to enhance reproducibility.

**Comment:** “Avoid the term ‘detailed genome-wide analysis of the FOX gene family’, because authors only used computational tools for this study.”

**Response:** In line with the feedback, we have replaced the term “detailed genome-wide analysis” with “comprehensive computational analysis” to more accurately represent our study’s scope.

We believe that these revisions have significantly improved the manuscript and have addressed all the concerns raised. We appreciate the reviewers’ constructive feedback and are hopeful that the revised manuscript meets the journal’s standards for publication.

Thank you for considering our revised manuscript. We look forward to your positive response.
