# Supplementary material for: Investigating the role of FOX gene family in development and stress response in Labeo rohita: A multi-faceted analysis of phylogeny and genome characterization
Source: PLoS One. 2025 Aug 21;20(8):e0323740. doi: 10.1371/journal.pone.0323740 (PMC12370200; doi:10.1371/journal.pone.0323740)
Supplement: Attachment — Submitted filename: Review comments 2.docx [file pone.0323740.s005.docx]

Dear Editor and Reviewers,

We would like to express our sincere gratitude for the time and effort you have dedicated to reviewing our manuscript entitled “**Investigating the Role of FOX Gene Family in Development and Stress Response in *Labeo rohita*: A Multi-faceted Analysis of Phylogeny and Genome Characterization**.” Your thoughtful and constructive feedback has been instrumental in improving the overall quality and clarity of our work.

Following the recent round of minor revision, we have carefully addressed the remaining comment provided by Reviewer #2, as detailed below:

**Response to Reviewer #2**

**Comment:**

Line no. 137–138: “These sequences were organized into 12 major clades...” But there are 11 clades. In Figure 1, they have also clustered the phylogenetic tree into 11 clades. Please rectify it.

**Response:**

The mentioned line has been corrected in the revised manuscript, changing the number of clades from 12 to 11 to accurately reflect the phylogenetic analysis and Figure 1. We have reviewed the manuscript thoroughly to ensure consistency throughout the text.

We appreciate your continued support and are hopeful that the manuscript is now suitable for acceptance. Thank you once again for your guidance and constructive suggestions throughout the review process.

Sincerely,

Shabana Naz

Department of Zoology, Government Sadiq College Women University, Bahawalpur 63100, Punjab, Pakistan.

Email: drshabananaz@gcuf.edu.pk
